# Does radiation therapy need more than two photon energies from Linac?

**DOI:** 10.3389/fonc.2022.1009553

**Published:** 2022-11-03

**Authors:** Xile Zhang, Fugen Zhou, Bo Liu, Tianyu Xiong, Xiangzhi Bai, Qiuwen Wu

**Affiliations:** ^1^ Image Processing Center, Beihang University, Beijing, China; ^2^ Department of Radiation Oncology, Peking University Third Hospital, Beijing, China; ^3^ Beijing Advanced Innovation Center for Biomedical Engineering, Beihang University, Beijing, China; ^4^ Department of Physics, Beihang University, Beijing, China; ^5^ Department of Radiation Oncology, Duke University Medical Center, Durham, NC, United States

**Keywords:** radiotherapy, linear accelerator, photon beam data characteristics, photo energy, energy synthesis

## Abstract

**Purpose:**

Modern Linacs are equipped with multiple photon energies for radiation therapy, and proper energy is chosen for each case based on tumor characteristics and patient anatomy. The aim of this study is to investigate whether it is necessary to have more than two photons energies.

**Methods:**

The principle of photon energy synthesis is presented. It is shown that a photon beam of any intermediate energy (E_syn_) can be synthesized from a linear combination of a low energy (E_low_) and a high energy (E_high_). The principle is validated on a wide range of scenarios: different intermediate photon energies on the same Linac; between Linacs from the same manufacturer or different manufacturers; open and wedge beams; and extensive photon energies available from published reference data. In addition, 3D dose distributions in water phantom are compared using Gamma analysis. The method is further demonstrated in clinical cases of various tumor sites and multiple treatment modalities. Experimental measurements are performed for IMRT plans and they are analyzed using the standard clinical protocol.

**Results:**

The synthesis coefficients vary with energy and field size. The root mean square error (*RMSE*) is within 1.1% for open and wedge fields. Excellent agreement was observed for British Journal of Radiology (BJR) data with an average RMSE of 0.11%. The 3D Gamma analysis shows a good match for all field sizes in the water phantom and all treatment modalities for the five clinical cases. The minimum gamma passing rate of 95.7% was achieved at 1%/1mm criteria for two measured dose distributions of IMRT plans.

**Conclusion:**

A Linac with two photon energies is capable of producing dosimetrically equivalent plans of any energy in-between through the photon energy synthesis, supporting the notion that there is no need to equip more than two photon energies on each Linac. This can significantly reduce the cost of equipment for radiation therapy.

## 1 Introduction

A modern linear accelerator (Linac) is often equipped with multiple photon energies. This versatility facilitates the choice of proper energy for each individual case. Different energy photon beams have different penetrating capabilities and other dosimetric properties. Lower energy photon beams (≤6MV) are often used to treat targets of shallow depths such as head and neck cancer and breast cancer, due to their requirement of limited penetration and sparing of distant organs. On the other hand, higher energy photon beams (≥10MV) are chosen for deep-seated tumors such as prostate cancer due to their higher penetration power and better skin-sparing ability (1). In order to have sufficient target coverage and reduce damage to surrounding critical organs, it is necessary to consider overall beam characteristics when choosing optimal photon energy (2). Therefore, the planner needs to decide the ideal beam energy when designing a treatment plan, and it may also vary with the gantry angle. Previous studies showed potential advantages of mixed energy photons in improving plan quality in prostate and breast plans (3–9). However, none of these studies have fully demonstrated the concept of photon energy synthesis systemaically and its potential advantages in radiation treatment.

Due to the complexity of the linear accelerator engineering and the requirement of dose rate output, the design of the accelerator only allows a limited number of photon energies (usually 2 to 3) to be available, such as 6MV to represent low energy and 15MV for high energy. Adding more energy levels will significantly increase the complexity and cost in engineering and manufacturing. In addition, it demands a premium in purchase and maintenance costs and increases the efforts in quality assurance to the user (10). Research efforts have been made to modify the Linac to produce more photon energies (11), but face serious challenges in clinical implementation and safety.

The aim of this study was to present a method of generating any equivalent photon energy from only two energies using a different approach. Instead of changing Linac design, this method focused on the observable and measurable effects of the photon beams, *i.e*., the dosimetric properties of the photon energy. The method was developed to synthesize a photon beam of known energy from the combination of other photon energies to meet the most stringent criteria. An immediate application of this technique is to deliver a radiotherapy treatment plan of an known energy photon beams on the same or another Linac with different photon energies to achieve near-identical dose distributions. This becomes convenient when that photon energy on one machine is unavailable unexpectedly, then the patient can still be treated, thereby preventing the treatment delay. The more profound benefit of this technique is that essentially any intermediate energy photon beam can be produced through the synthesis, so there is no need to have more than two photon energies on a medical Linac. This will have a large effect on Linac manufacturers in that complex engineering design on waveguide to accommodate more photon energies becomes unnecessary; also on the end users on saving the cost of purchase, commissioning, maintenance, and quality assurance.

In the following sections, the principle of photon energy synthesis is first presented. It is shown that any middle energies can be produced with equivalent dose characteristics from a low energy and a high energy. Both percent depth dose (PDDs) and off-axis profiles (or off-center ratios, OCRs) were included to improve the consistency of the synthesized and actual photon energies. The method was then validated on a wide range of scenarios: different intermediate energies on the same Linac; between Linacs from the same manufacturer or different manufacturers; non-flat beams such as wedges; and all photon energies available from previously published reference data. The accuracy of the photon synthesis was evaluated quantitatively for each scenario. In addition, 3D dose distributions from standard water phantom and actual patient CTs were compared using Gamma analysis (12). The method was further demonstrated in a few clinical cases of various tumor sites and with different treatment modalities (3DCRT, IMRT, and VMAT). Finally, experimental measurements were performed by delivering IMRT plans to QA devices of planar detector arrays, and analyses were performed using the standard protocol. Additional benefits and drawbacks were discussed subsequently.

## 2 Materials and methods

### 2.1 Photon energy synthesis method

In radiotherapy, the penetrating ability of a photon beam is mainly a function of the mean photon energy, usually expressed by its central axis PDD characteristics in water. Beam energy also influences the OCR shape of both inside and outside the field boundaries, owing to phantom scatter, leakage and scatter from the collimator system including wedge and flattening filters etc.

The PDD and OCR are two important characteristics of photon beams associated with energy. For a given Linac equipped with multiple photon energies, including one low energy (*E_low_*), one high energy (*E_high_*), and optional energies in the middle (*E_mid_*), the PDDs and OCRs (at different depths) of commissioning beam data for each energy were used for photon energy synthesis. Considering the simplicity and practicability of the synthesis method, a simple least square fitting method was used. For each geometry, *i.e*., field size, synthetic photon energy (*E_sy_*_n_) was formed as a linear combination of *E_low_* and *E_high_* with its corresponding PDDs and OCRs expressed as:


(1) $$\left\{ \begin{array}{l} PDD_{E_{syn}}=\alpha \cdot PDD_{E_{low}}+\beta \cdot PDD_{E_{high}}\\ OCR_{E_{syn}}=\alpha \cdot OCR_{E_{low}}+\beta \cdot OCR_{E_{high}} \end{array}\right.$$

where *α* and *β* are the weighting factors for *E_low_* and *E_high_*. To match *E_syn_* to another actual photon energy (*E_mid_*) with commissioning beam data, a linear least-squares fitting method is performed to derive the coefficient matrices (*α, β*) which minimize the weighted sum of the root mean squared error (*RMSE*) for PDDs and OCRs for each beam geometry:


$$\underset{\alpha ,\beta }{\arg\min}\left(RMSE_{PDD}+RMSE_{OCR}\right)$$

(2) $$RMSE_{PDD}=\sqrt{\frac{1}{m}\sum_{i=1}^{m}{\left(PDD_{E_{mid}}(i)-PDD_{E_{syn}}\left(i\right)\right)}^{2}}$$

$$RMSE_{OCR}=\sqrt{\frac{1}{n}\sum_{i=1}^{n}{\left(OCR_{E_{mid}}(i)-OCR_{E_{syn}}\left(i\right)\right)}^{2}}$$

Here, *m* and *n* represent the total number of data points of PDD and OCR curves. All PDDs and OCRs data were processed before the fitting. The PDD data were sampled at 1 mm spacing and were normalized to 100% at *d_max_*, the depth of maximum dose, and the OCR data were sampled at 1 mm spacing and were normalized to central axis value that is the same as PDD at that depth.

The synthesis is performed directly on the measured commissioning data and, thus is independent of the treatment planning system (TPS) or dose calculation algorithms. Therefore, the result of the synthesis (coefficient matrices for different beam geometries) can be applied to treatment plans directly and outside of the TPS.

### 2.2 Beam data used for validation

A series of beam data were collected to evaluate the photon energy synthesis method. At first, the full set of beam data of three different Linacs were used to demonstrate the principle under different scenarios. The three Linacs are: a TrueBeam Linac and a Clinac 2300iX Linac from Varian (Varian Medical Systems, Palo Alto, CA), and an Elekta Infinity Linac (Elekta Oncology Systems, Crawley, UK). Then, standard photon reference beam data of a wide range of energies published in British Journal of Radiology (BJR) Supplement 25 (13) was used to demonstrate the generality of the principle.

The beam data of multiple energies were acquired in the water tank, including both open field and wedge field under different geometries, summarized in **Table 1**. PDDs data were from 0 to 30 cm in depth, and OCRs were at four different depths (depths of 5, 10, 20, and 30 cm) with 100 cm source to surface distance (SSD). These measured beam data were used for commissioning the beam calculation model of the planning system, which meets the manufacturer’s full specifications. These Linacs beam data were acquired at different times and in different facilities.

**Table 1:** Geometries of the Linacs beam data with multiple energies.

Data Source	Field Type	Energy (MV)	Beam Data	Field Size (cm^2^)	Measurement device
Varian TrueBeam	Open Field	6, 8, 10, 15	PDD, OCR	3×3, 4×4, 6×6, 8×8, 10×10, 20×20, 30×30, 40×40	CC13
	Wedge Field	6, 10, 15	PDD, OCR	4×4, 10×10, 20×20, 30×30, 30×40	CC13
Clinac 2300iX	Open Field	6, 15	PDD, OCR	3×3, 4×4, 6×6, 8×8, 10×10, 20×20, 30×30, 40×40	CC13
Elekta Infinity	Open Field	10	PDD, OCR	3×3, 4×4, 6×6, 8×8, 10×10, 20×20, 30×30, 40×40	CC13
BJR Data (13)	Open Field	4, 5, 6, 8, 10, 12, 15, 18, 21	PDD	4×4, 5×5, 6×6, 7×7, 8×8, 9×9, 10×10, 12×12, 15×15, 20×20, 25×25, 30×30, 35×35, 40×40	Unknown

CC13 is the compact cylindrical ion chamber with a collection volume of 0.13 cc used in water phantom scanning.

### 2.3 Photon energy synthesis examples

#### 2.3.1 On the same Linac

Firstly, the photon energy synthesis method was validated using the Varian TrueBeam golden beam data which is the average of three TrueBeam machines beam data at one institution (14). Based on 6MV and 15MV beam data, 8MV and 10MV beam data were synthesized and compared with the directly measured data at different open field sizes. For the wedge field, due to the introduction of the wedge plate, the situation of the wedge field is different, in that the profiles are no longer flat, and PDD curves of the wedge field also change slightly from the open field. Therefore, 6, 10, and 15MV of 30° wedge beam data were selected to evaluate the photon energy synthesis method for the wedge field.

For each field size, PDD was divided into build-up region (depth < *d*_max_) and descending region (depth > *d*_max_), and OCR was divided into the three regions (in-field, penumbra, and out-field) which have been described in the AAPM report (15). A point-by-point dose difference was compared in each region between *E_syn_* and *E_mid_*. To quantitatively evaluate the residual error, in addition to the *RMSE*, 1D-Gamma analysis using criteria of dose difference (DD) of 2% or 1% and distance to agreement (DTA) of 1 mm were performed. Furthermore, the dosimetric characteristic parameters (DCPs) including surface dose (*PDD_0_*), *PDD_10_* (PDD at 10 cm depth), *PDD_20_*, *d_80_* (depth where PDD=80%), *d_50_*, and *d_max_* were also analyzed and compared.

#### 2.3.2 Between Linacs from the same manufacturer

For Linacs from the same manufacturer, slight differences in PDD and OCR exist due to small design variations of the Linac head (16). To explore energy synthesis between Linacs from the same manufacturer, 6MV and 15MV photon beam data of Varian Clinac 2300iX Linac (IX-6MV and IX-15MV) were used to match the Varian TrueBeam Linac 10MV photon beam data (TB-10MV). The rationale is that, if the match result is acceptable, then a treatment plan of specific energy can be delivered on another Linac with different photon energies to achieve the same dose distributions. The IX-6MV and IX-15MV were used as *E_low_* and *E_high_*, TB-10MV as *E_mid_*, respectively. Only open fields are considered in this example, and the analysis method and parameter comparison are performed the same way as in the previous section (2.C.1).

#### 2.3.3 Between Linacs from different manufacturers

Commonly, there are large differences in PDDs and OCRs for Linacs from different manufacturers, which may be resulted from significantly different designs of the head and related components, as well as changes in the bending magnet impacting the incident electron source width (17). It is expected that the quality of the energy synthesis may be worse than those from the same manufacture, or the results may not be clinically acceptable at all. Therefore, it will be interesting to explore the possibility of energy synthesis between Linacs from different manufacturers. Here tests were performed to synthesize 10 MV photon beam data of Elekta Infinity Linac (EI-10MV) using Varian TrueBeam Linac 6 MV and 10 MV beam data (TB-6MV and TB-15MV).

#### 2.3.4 Photon energy synthesis of BJR data

Furthermore, the generality of the method was tested using the beam data published in the British Journal of Radiology supplement (BJR supplement 25) (13), which contains reference photon beam data for a variety of photon energies used in radiation therapy. One low photon energy (4MV) was chosen as *E*_low_ and one high photon energy (21MV) was chosen as *E*_high_ as the basis for the synthesis. All other photons (6MV, 8MV, 10MV, etc.) are synthesized using these two and compared with the one in the publication. Then, *E*_low_ was changed to 6MV while *E*_high_ was fixed at 21MV, and the synthesis process was repeated for all energies between the new *E*_low_ and *E*_high_. The process was repeated until *E*_low_ reaches 21MV. Because OCRs data were not available from the publication, only PDDs data was used in the fitting. The beam data were processed and converted to the same format before the fitting.

#### 2.3.5 Validation in the water phantom

Evaluations on the synthesis were performed so far on the limited number of data points, either along or perpendicular to the depth axis, *i.e.*, essentially in one-dimensional space. To comprehensively evaluate the method in 3D geometry and clinical cases, the treatment planning system (TPS) is needed. The TrueBeam golden beam data and the Varian Clinac 2300iX Linac beam data were imported into the Varian Eclipse TPS (version 15.5) and used to configure the anisotropic analytical algorithm (AAA) dose calculation engine following the manufacturer’s instructions. A water phantom with a size of 60 × 60 × 60 cm^3^ was used for all calculations, and two sets of test plans with open-field beams of 3 × 3, 4 × 4, 6 × 6, 8 × 8, 10 × 10, 20 × 20, 30 × 30 and 40 × 40 cm^2^ field sizes were also created. In the first set, each plan has one beam of different field sizes using the actual TB-10MV photon with 1000 MUs. For the second set, each plan has two beams of the same geometry but different energies (TB-6MV and TB-15MV, or IX-6MV and IX-15MV) and MUs. The MU of each beam was determined based on the coefficients (*α_i_, β_i_*) of the energy synthesis performed in sections 2.C.1 or 2.C.2 and output factors. Therefore, the second set of plans are for the synthesized TB-10MV photons. The dose for the two sets of plans was calculated using the AAA algorithm at a dose grid of 1×1×1 mm^3^, and the 3D dose matrices were exported in DICOM format for 3D Gamma analysis using the Plastimatch toolkit (18). Since both dose matrices share the same coordinate system from the water phantom in TPS, the DTA criteria was set at a low value of 1 mm. The dose difference criteria were varied from 1% (most strict) to 2%. The dose threshold is set at 5% (below which values are mostly out-of-field points).

#### 2.3.6 Clinical case treatment plans

The practicality of the photon energy synthesis in clinical treatment was investigated based on treatment plans of five patient cases with targets at different anatomic locations, including intra-cranial, lung, breast, liver, and prostate. At first, three plans of 3DCRT, IMRT, and VMAT were generated for each case using the actual TrueBeam 10MV photon. A 120-millennium multileaf collimator (MLC) was used for beam modeling and the sliding window technique was used for IMRT and VMAT. All plans were optimized to meet the clinical requirements. Then, plans with synthesized TrueBeam 10MV photon were generated. For each plan, each beam was duplicated with one beam of the TrueBeam 6MV and the other with 15MV photon. Similarly, the MUs were determined by the coefficients (*α_i_, β_i_*) of the energy synthesis performed in section 2.C.1 and output factors. The dose of all plans was also computed using the AAA algorithm and the dose grid at 1×1×1 mm^3^. The comparisons were performed using 3D Gamma analysis, the dose-volume histograms (DVHs), and isodose displays on patient CT images.

#### 2.3.7 Clinical case measurement verification on QA device

To ensure the actual delivery accuracy of the synthetic photon energy, IMRT plans of the five patient cases presented in the previous section (2.C.6) were verified through the measurements, by compuing the differences between two sets of measured data, and between measurement and calculations. The IMRT plans were projected on CT images of the 2D ionization chamber array (MatriXX, IBA, Schwarzenbruck, Germany). The AAA algorithm is used in calculation the dose, and the dose grid is 1×1 mm^2^. The 2D dose plane of the effective measurement area of the verification plan was exported with a resolution of 0.47 mm and imported into the software myQA (version 2.10, IBA, Schwarzenbruck, Germany). IMRT plans with the actual TrueBeam 10MV photon and synthesized 10MV photon (6 & 15 MV) beams are delivered to the QA device. Global 2D Gamma analysis was performed between measured and calculated, and among measured dose distributions (12). The dose threshold is set at 5%. The dose difference criteria are varied from 1% to 3%. The DTA criteria is set at 1 mm or 2 mm. The maximum and mean dose differences are also evaluated.

## 3 Results

### 3.1 On the same Linac

#### 3.1.1 Open field

**Figure 1:**
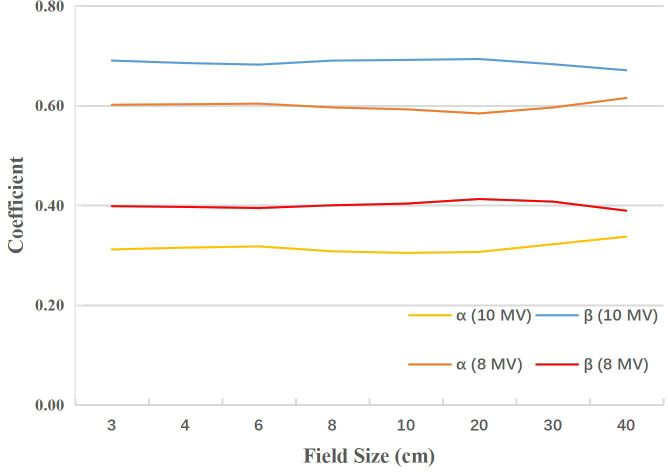
The synthesis coefficients (*α, β*) as a function of field size for E_syn_-8MV and E_syn_-10MV.

The synthesis coefficients (*α, β*) for E_syn_-8MV and E_syn_-10MV of different field sizes from the data fitting based on both PDDs and OCRs were shown in **Figure 1**. They are quite different between E_syn_-8MV and E_syn_-10MV, also vary slightly with field size. The *RMSE* values of PDDs and OCRs for E_syn_-8MV and E_syn_-10MV were presented in **Table 2**. For each field size, the maximum *RMSE* was no more than 1.0%.

**Table 2:** The RMSE of PDDs and OCRs between *E_mid_* and *E_syn_* for 8MV and 10MV.

Energy		Square Field Size (cm)							
			3	4	6	8	10	20	30	40
8-MV	*RMSE_PDD_*		0.64	0.65	0.63	0.65	0.64	0.76	0.87	0.96
	*RMSE_OCR_*		0.30	0.34	0.34	0.38	0.45	0.47	0.48	0.70
10-MV	*RMSE_PDD_*		0.46	0.51	0.50	0.50	0.52	0.58	0.77	0.85
	*RMSE_OCR_*		0.10	0.12	0.16	0.16	0.22	0.27	0.34	0.39

(Unit: %).

Taking 10 MV energy synthesis as an example, **Table 3** summarizes the 1D-Gamma analysis result. At the 1%/1mm criterion, the passing rate decreases with increasing depth of larger field, and most PDDs and OCRs achieved 100% passing rate at the 2%/1mm criterion except for a few OCRs at the 300 mm depth.

**Table 3:** 1D Gamma passing rate (%) for PDD and OCR between E_mid_ and E_syn_ (10MV).

		Square field size (cm)							
Type	Depth (cm)	3	4	6	8	10	20	30	40
OCR	5	100	100	100	100	100	100	100	100
	10	100	100	100	100	100	100	100	100
	20	100	100	100	100	100	79.4/100	71.7/100	74.8/100
	30	100	100	100	89.2/100	81.7/100	56.7/86.1	59.3/81.5	47.3/83.2
PDD		95.3/99.7	94.7/99.7	94.4/99.7	95.0/99.7	94.0/99.7	99.7/99.7	99.7/99.7	97.7/99.7

The criteria were first set at 1%/1mm, if γ is not 100%, then a relaxed criteria of 2%/1mm was used and the new γ is shown as the second number in each cell.

**Figure 2:**
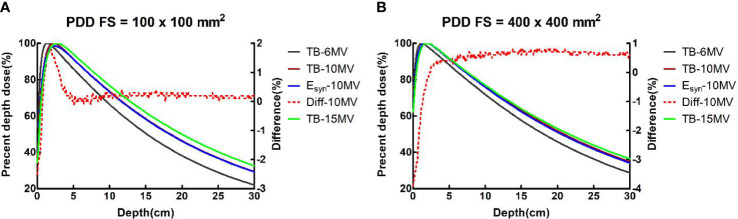
TB-6MV, TB-10MV, E_syn_-10MV and TB-15MV PDDs and differences between TB-10MV and E_syn_-10MV for the **(A)** 100 × 100 mm^2^ and **(B)** 400 × 400 mm^2^ field size.

**Figure 3:**
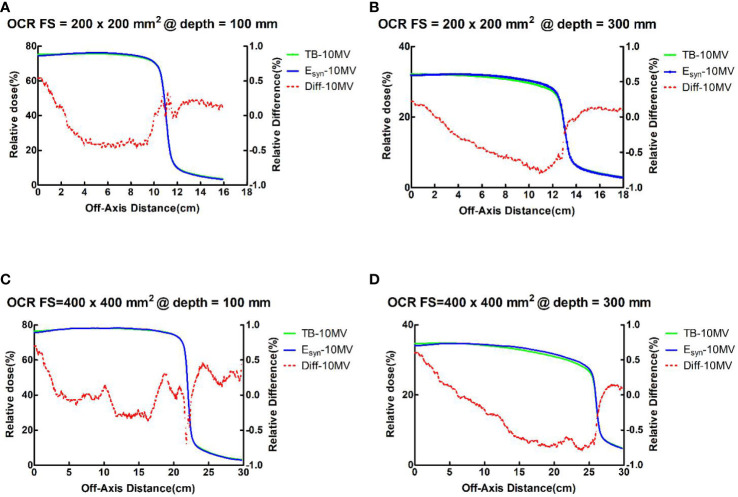
TB-10MV and E_syn_-10MV OCRs and differences for the 200 × 200 mm^2^ field size at depth = **(A)** 100mm and **(B)** 300mm, and for the 400 × 400 mm^2^ field size at depth of **(C)** 100mm and **(D)** 300mm.

For PDDs at all field sizes, the maximum dose difference between TB-10MV and E_syn_-10MV was no more than 3.9% at the build-up region and 1.3% at the descending region. Dose differences were within 1.4% in three regions for OCRs at different depths. **Figures 2**, **3** compare the dose differences of the TB-10MV and E_syn_-10MV for 200 × 200 mm^2^ and 400 × 400 mm^2^ field size. Additional comparisons of other dosimetric parameters are summarized in **Table S1** (in the supplementary document).

#### 3.1.2 Wedge field

For the wedge field, the OCR is not flat and the gradient is large compared to the open field, which can be challenging for photon synthesis. The *RMSE* values of PDDs and OCRs for E_syn_-10MV were summarized in **Table S2** (in the supplementary document). The *RMSE* value of PDDs and OCRs for the wedge field was slightly higher than the open field with a maximum RMSE of 1.1%. Differences in DCPs in the wedge field were also larger than in the open field. The result showed all but *PDD_0_* agree within 1.2% or 4 mm.

**Figure 4:**
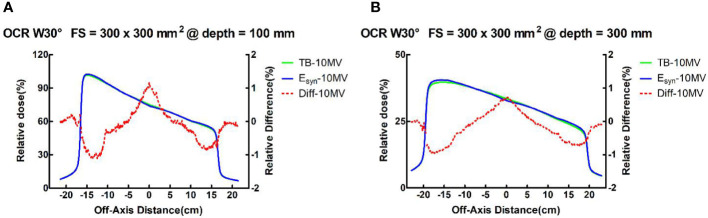
TB-10MV and E_syn_-10MV OCRs and differences for the wedge 30°field at size of 400 × 400 mm^2^ at depths of **(A)** 100mm and **(B)** 300mm.

For all field sizes, the differences between TB-10MV and E_syn_-10MV in the wedge field were no more than 3.9% in the build-up region and 1.7% in the descending region for PDD. The OCR differences in all depths were within 1.2% in the three regions. The results for small field size were better than large field size. **Figure 4** shows the differences of PDD and OCR between TB-10MV and E_syn_-10MV for 30° wedge in 300 × 300 mm^2^ field size.

For the 1D Gamma analysis, a 100% passing rate (2%/1mm) was achieved for most of PDDs and OCRs. More details can be found in **Table S3** of the supplementary document.

### 3.2 Between Linacs from the same manufacturer

For the energy synthesis between Varian Truebeam and Clinac 2300iX Linacs, the maximum *RMSE* of PDDs and OCRs was 0.9% (**Table S4**). The maximum difference was 9.3% in the build-up region and 1.4% in the descending region for PDDs. The OCR differences in the three regions were within 2.4% and the maximum difference was 2.1% for the in-field region. All DCPs except for *PDD_0_* agreed within 1% or 2 mm for field size less than 30 × 30 cm^2^.

For the 1D-Gamma analysis, most of PDDs and OCRs achieved a 100% passing rate at the 2%/1mm criterion (See **Table S5** for more details).

### 3.3 Between Linacs from different manufacturers

For energy synthesis between Varian Truebeam and Elekta Infinity Linacs, the maximum *RMSE* of PDDs and OCRs was 0.76% (See **Table S6** for more details). The maximum variations were less than 4.1% in the build-up region and 1.3% in the descending region for PDDs. The maximum difference of OCRs was no more than 3.1% in the in-field region for all field sizes. This is probably due to the different design of the flattening filter in the linacs. All DCPs but *PDD_0_* agreed within 1% or 2 mm for all filed sizes. For *PDD_0_*, the maximum difference was 4.1% for all field sizes.

The 1D Gamma passing rates of OCRs were more than 90% at the 2%/1mm criterion for most filed sizes, with the minimum passing rate of 85.5%. Most of the PDDs achieved a 100% passing rate at the 2%/1mm criterion (**Table S7**).

### 3.4 Photon energy synthesis of BJR data

The differences of PDDs between E_mid_ and E_syn_ for the 10 × 10 cm^2^ field size is presented in **Figure S1** (supplementary document), with E_high_ was fixed at 21MV and when E_low_ was changed to 4MV, 5MV, 6MV, 8MV, 10MV, 12MV, and 15MV. Good agreement was observed between the PDDs of *E_mid_* and *E_syn_*. The average *RMSE* value for all energies and field sizes is 0.11%. The maximum *RMSE* value is 0.22% for the 10MV 5 × 5 cm^2^ field size synthesized from 4MV and 21MV. **Table 4** lists the *RMSE* of multiple syntheses using different E_low_ energies for the 10 × 10 cm^2^ field size.

**Table 4:** RMSE (%) of different synthetic energy for the 100 × 100 mm^2^ field with 21 MV as *E_high_*.

E_mid_/E_low_	5	6	8	10	12	15	18
**4**	0.09	0.17	0.20	0.20	0.18	0.14	0.08
**5**	–	0.11	0.16	0.18	0.16	0.14	0.08
**6**	–	–	0.11	0.14	0.14	0.13	0.08
**8**	–	–	–	0.07	0.09	0.09	0.07
**10**	–	–	–	–	0.05	0.07	0.06
**12**	–	–	–	–	–	0.06	0.05
**15**	–	–	–	–	–	–	0.05

### 3.5 Validation in the water phantom

**Figure 5:**
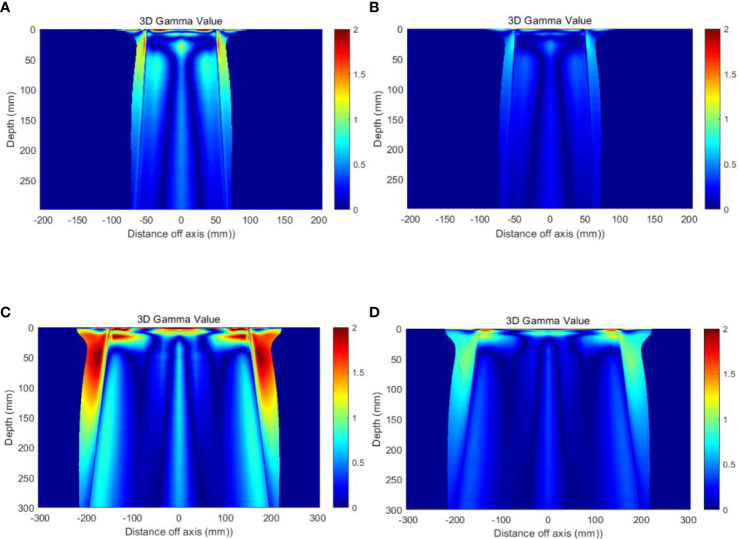
Gamma distribution on the central planes **(A)** Field size 10 × 10 cm^2^, with 1%/1mm criteria **(B)** Field size 10 × 10 cm^2^, with 2%/1mm criteria **(C)** Field size 30 × 30 cm^2^, with 1%/1mm criteria and **(D)** Field size 30 × 30 cm^2^, with 2%/1mm criteria.

**Table 5** presents the results of 3D Gamma analysis for 3D dose matrices using a 5% threshold value and 2%/1mm and 1%/1mm criteria. **Figure 5** shows the Gamma distribution in the central planes for each group of plans with TB-10MV and E_syn_-10MV photon beams. The points that failed the criteria of 1%(DD)/1mm(DTA) were mainly located in the shallow surface region or out-filed area, as shown in **Figures 5C, D**.

**Table 5:** Gamma analysis results for the open fields between TB-10MV and E_syn_-10MV photon beams.

Square Field Size (cm) Criteria	3	4	6	8	10	20	30	40
1%/1mm	92.4	88.9	90.6	96.4	96.6	89.9	91.3	57.6
2%/1mm	99.4	98.8	99.4	99.3	99.3	99.0	99.0	95.0

### 3.6 Clinical cases

**Figure 6:**
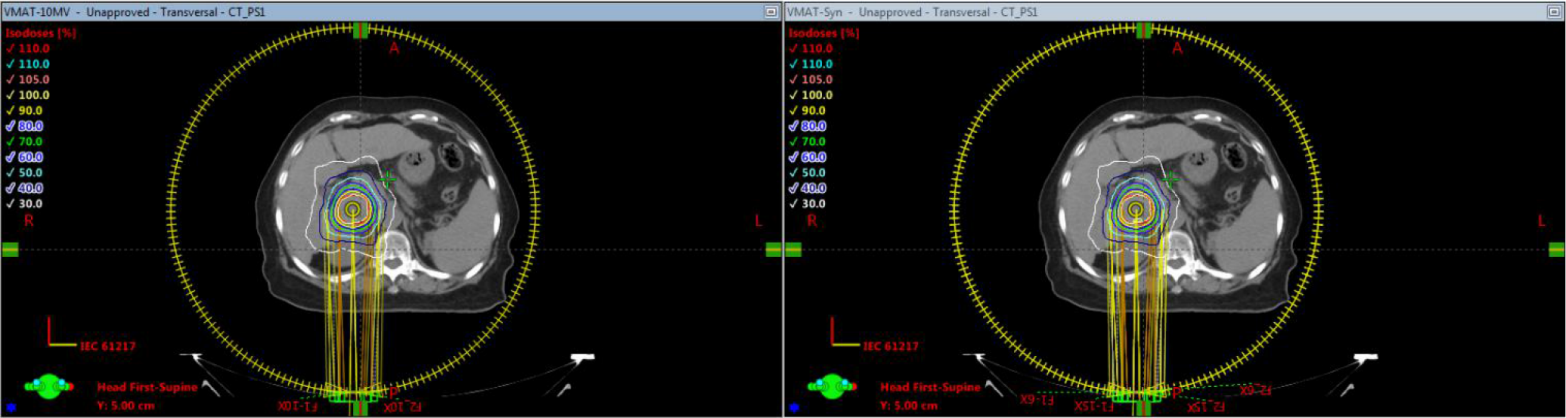
Comparison of dose distributions of VMAT plan for Case 2 (Left: TB-10MV; Right: E_syn_-10MV).

**Figure 7:**
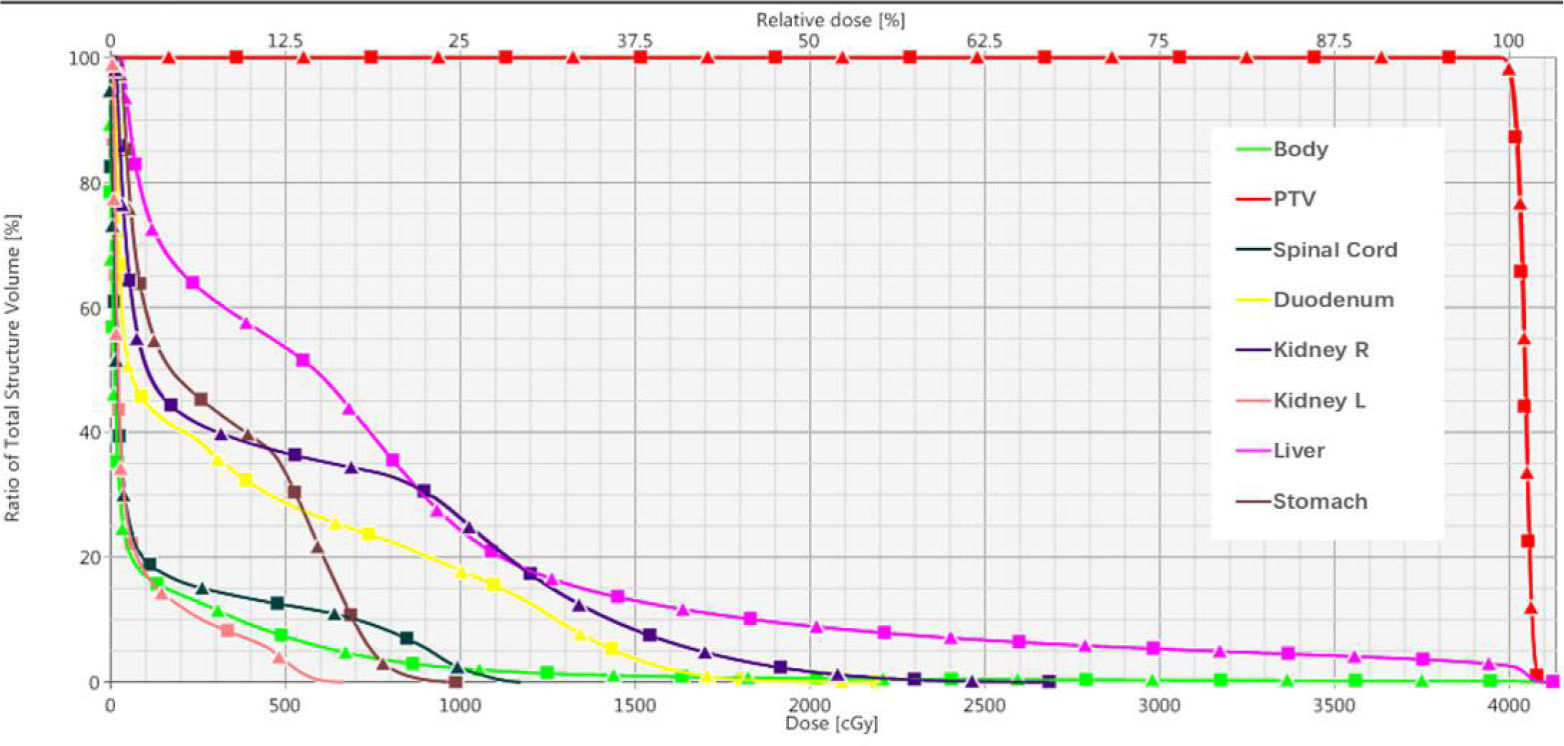
Dose-volume histograms of corresponding VMAT plan in Case 2. (Square solid line: TB-10MV; Triangle solid line: E_syn_-10MV).

**Table 6** summarizes the results of 3D-Gamma analysis for five anatomic sites using 1%/1mm criteria with a 5% dose threshold. Excellent passing rates were observed for all cases with a minimum gamma passing rate of 97.4%. Through visual examination, good agreements of iso-dose lines and DVHs were observed for all plans. For details about the dose distributions and DVHs of two corresponding plans for each case, see **Figures S2-S11** in the supplementary document). As an example, **Figure 6** presents isodose distributions of two corresponding VMAT plans with two full arcs in the liver case, and **Figure 7** shows the similarity between DVH lines.

**Table 6:** 3D Gamma passing rate (%) of 1%/1mm for clinical cases. Criteria = 1%/1mm.

Modality	3D-CRT	IMRT	VMAT
Case 1 (breast)	97.7	97.4	99.1
Case 2 (liver)	100.0	100.0	100.0
Case 3 (prostate)	99.5	99.6	99.0
Case 4 (lung)	99.6	99.6	99.7
Case 5 (intra-cranial)	99.9	100.0	100.0

### 3.7 QA verification of IMRT plans of clinical cases

The maximum dose differences were within 5.3% and the mean dose differences were no more than 0.2% between two measured dose distributions of IMRT plans with the actual and synthesized 10MV photon. **Table 7** presents the results of 2D-Gamma analysis for clinical cases IMRT plans verification. High passing rates were obtained for the IMRT plans of each clinical case. The minimum gamma passing rate of 98.7% using 2%/1mm criteria for two measured dose distributions of each pair of IMRT plans.

**Table 7:** 2D Gamma passing rate (%) for IMRT plan verification of five clinical cases (actual TB-10MV measurement vs synthesized 10MV measurement).

	Actual TB-10MV measurement vs synthesized 10MV measurement	Actual TB-10MV measurement vs. IMRT plan	Synthesized 10MV Measurement vs. IMRT plan
Criteria	1%/1mm	2%/2mm	2%/2mm
Case 1 (breast)	95.7	96.2	99.8
Case 2 (liver)	100.0	98.4	100.0
Case 3(prostate)	98.9	100.0	97.7
Case 4 (lung)	98.7	100.0	99.3
Case 5 (intra-cranial)	100.0	100.0	100.0

## 4 Discussion

In this study, we developed a method to synthesize a photon beam of known energy from the combination of other photon energies from a linear accelerator. With this synthetic technique, we can match a given known energy photon beam characteristics under the most stringent criteria. This method is fundamentally different from the traditional energy match where modifications are needed on the design of accelerator waveguide or beam tuning are performed. This method took alternative approach by focusing on the observable physical effects such as measurable dosimetric quantities that are commonly accepted by the community to characterize photon energy.

It is worthy to point out that, many examples in this study were used to demonstrate, the *equivalence* but not the *superiority*, of the synthetic energy (E_syn_) photon beam to the actual photon beam, in their dosimetric properties and treatment plans on patient cases in different modalities. Only after the equivalence is proven and shown, we can then infer that any continuous photon energies can be synthesized in such fashion and they include those photon energies that do not exist or are available currently on Linac or TPS, *i.e*, they have not been attempted previously in any radiotherapy treatment plans. We further speculate that they may offer some potential dosimetric and clinical benefits over the plans with current available photon energies.

There are many potential clinical benefits of this photon synthesis technique. For example, a radiotherapy treatment plan of known photon beams can be delivered on the same or another Linac with different photon energies to achieve near-identical dose distributions. This becomes convenient when that photon energy on a machine is down unexpectedly, then the patient can still be treated on the same Linac or a different Linac, provided that other photon energies are still available and the synthesis has been performed and judged acceptable beforehand, thereby preventing the patient treatment delay.

One apparent conclusion from this study is that, there is no need to have more than two photon energies on a medical Linac because any intermediate energy photon beam can be produced through the synthesis. This will have a large effect on the manufacturer that complex engineering design on waveguide to accommodate more photon energies becomes unnecessary; instead, focus can be placed on improving the efficiency and output of the existing photon energies. For the users at the clinic, this should also have a large cost saving, in the initial purchase, commissioning, maintenance, and quality assurance.

Another potential benefit of this technique is that the synthesized photon energy can be continuously varying between E_low_ and E_high_, and many of them are not available currently. Therefore this can be a useful new tool in the treatment planning, although the physical and clinical benefits will need to be explored. For example, the method can be used to synthesize a photon energy of 8MV-FFF based on 6MV-FFF and 10MV-FFF.

It is worth to point out that one advantage of this method is that the synthesis is performed on the commissioning beam data collected in water phantom, therefore, the result is independent of the treatment planning system or different photon dose calculation algorithms, i.e., it can be applied to any treatment plans. Also as demonstrated, this is applicable to a wide variety of scenarios – open and wedge field plans; Linacs from different manufacture; 3DCRT, IMRT, and as well as VMAT.

Certainly, there are shortcomings of this synthesis method. One obvious limitation is the delivery efficiency – each field using the E_syn_ needs to be delivered twice using E_low_ and E_high_. This may not become a serious issue because the radiation delivery is taking less and less portion of the time slot compared with other steps such as patient setup and image guidance, the overall effect of this double radiation delivery may be insignificant. Another limitation is the relatively poorer match results at the surface and the out-of-field regions, even though they may not be significant as demonstrated in the 3D gamma analysis, the overall dose distributions should be carefully evaluated for each individual case. In addition, the differences in other beam delivery modification devices were not taken into the consideration of the synthesis, such as the multi-leaf collimator, of which the design can vary from manufacture to manufacture, and a treatment plan with certain type of MLC may not be easily converted to another type, and separate validations are necessary for clinical implementation. For the cases with same type of MLC, very small differences were observed in our limited cases. It is known that dose distributions of IMRT and VMAT plans depend on the MLC parameters such as leaf transmission and dosimetric leaf gap, which are photon energy dependent. However, they are monotonically changing with energy, negligible differences were observed in our plan comparisons and QA measurement due to the “interpolation” nature of the energy synthesis.

Granted, a E_syn_-10MV photon beam is not the same as true 10MV photon beam. They are equivalent because the measurable dosimetric effects are the same. For example, their energy spectrums are different – the maximum photon energy is 10MeV in the true 10MV photon beam, while the maximum photon energy in E_syn_-10MV is the same as in E_high_ used for the synthesis, so it can be 15MeV or 18MeV. It is possible that there may exist differences in some other quantities. One example is the neutron production once the photon energy exceeds 10 MV (19), which may lead to an increase in the incidence of secondary cancer after photon radiotherapy. The effects of neutron radiation dose are fairly complex and commonly not available in many photon treatment planning system, and was not included for evaluation in this study. Recent studies have shown that the high-energy neutron component may have been overestimated in the past (20). The American Association of Physicists in Medicine (AAPM) TG 158 document clearly states that there is a trade-off between high and low energy photon in neutron production and higher stray photon dose (21).

## 5 Conclusions

We have proposed a method to synthesize photon beams of known energy from the combination of existing photon energies from a medical linear accelerator. The synthesis produces excellent agreement to meet the most stringent criteria. Comprehensive evaluation of the method was performed under a wide range of scenarios: from open to wedge beams, from single to multiple manufactures, from 3DCRT to VMAT. The generality of the synthesis principle was verified using the published reference beam data. The technique was applied to a variety of clinical cases with different modalities and experimentally verified on QA devices. Therefore, the answer to the title question of whether there is need of more than two photon energies for radiation therapy is no. This can save the initial expenses and subsequent maintenance costs to the users. This technology also implies that effectively any continuous adjustable photon energy can be used in radiation treatment planning, thereby giving the planner another degree of freedom in creating optimal treatment plans.

## Data availability statement

The datasets generated during and/or analyzed during the current study are available from the first author on reasonable request.

## Author contributions

XZ organized and performed photon energy synthesis study experimental design, data collection, curation, data analysis, and interpretation. He was responsible for writing and revising the manuscript. FZ participated in the study design and data interpretation. BL initiated the project and participated in the study design, data analysis, and revising the manuscript. TX collated some experimental data. XB participated in part of the research design. QW initiated the project and participated in study design, interpretation, and revising the manuscript. All authors read and approved the final manuscript.

## Funding

This work was supported by the National Key Research and Development Program of China under Grant 2019YFB1311300 and 2019YFB1311301, and the Fundamental Research Funds for the Central Universities.

## Conflict of interest

The authors declare that the research was conducted in the absence of any commercial or financial relationships that could be construed as a potential conflict of interest.

## Publisher’s note

All claims expressed in this article are solely those of the authors and do not necessarily represent those of their affiliated organizations, or those of the publisher, the editors and the reviewers. Any product that may be evaluated in this article, or claim that may be made by its manufacturer, is not guaranteed or endorsed by the publisher.
