# Supplementary material for: Does radiation therapy need more than two photon energies from Linac?
Source: Front Oncol. 2022 Nov 3;12:1009553. doi: 10.3389/fonc.2022.1009553 (PMC9669309; doi:10.3389/fonc.2022.1009553)
Supplement: Supplementary file 1 [file DataSheet_1.docx]

1. **Dosimetric Characteristics Parameters (DCP)s for open field.**

Table S1. Difference of PDDs between TB-10 MV and E_syn_-10MV in open field on the same Varian Truebeam Linac. The PDD*_x_* is PDD at depth of *x* mm, so PDD_0_ is at surface, and PDD_100_ at 100 mm depth. The d*_y_* is depth at PDD value of *y*, so dmax is depth of maximum dose, and d_80_ is depth of PDD of 80% (beyond dmax).

| DCP | Square Field Size  （mm） | 30 | 40 | 60 | 80 | 100 | 200 | 300 | 400 |
| --- | --- | --- | --- | --- | --- | --- | --- | --- | --- |
| *PDD_0_ (%)* | TB-10MV | 30.0 | 30.7 | 33.0 | 35.5 | 37.6 | 49.4 | 57.5 | 62.2 |
|  | E_syn_-10MV | 32.4 | 33.2 | 35.4 | 37.8 | 40.1 | 52.3 | 61.1 | 66.1 |
|  | Diff | -2.4 | -2.5 | -2.4 | -2.3 | -2.5 | -2.9 | -3.6 | -3.9 |
| *PDD_100_ (%)* | TB-10MV | 69.9 | 70.7 | 71.9 | 73.0 | 73.5 | 75.1 | 75.9 | 76.5 |
|  | E_syn_-10MV | 69.8 | 70.8 | 72.0 | 72.9 | 73.2 | 75.6 | 75.2 | 75.8 |
|  | Diff | 0.1 | -0.1 | -0.1 | 0.1 | 0.3 | 0.5 | 0.7 | 0.7 |
| *PDD_200_ (%)* | TB-10MV | 42.1 | 42.8 | 44.2 | 45.5 | 46.4 | 49.3 | 50.8 | 51.8 |
|  | E_syn_-10MV | 42.1 | 42.8 | 44.1 | 45.4 | 46.1 | 48.9 | 50.1 | 51.0 |
|  | Diff | 0.0 | 0.0 | 0.1 | 0.2 | 0.3 | 0.4 | 0.7 | 0.8 |
| *d_max_ (cm)* | TB-10MV | 2.3 | 2.4 | 2.5 | 2.4 | 2.4 | 2.2 | 2.1 | 2.0 |
|  | E_syn_-10MV | 2.4 | 2.5 | 2.6 | 2.5 | 2.5 | 2.1 | 2.0 | 1.8 |
|  | Diff | -0.1 | -0.1 | -0.1 | -0.1 | -0.1 | 0.1 | 0.1 | 0.2 |
| *d_80_ (cm)* | TB-10MV | 7.3 | 7.5 | 7.8 | 8.0 | 8.1 | 8.4 | 8.6 | 8.8 |
|  | E_syn_-10MV | 7.4 | 7.6 | 7.9 | 8.0 | 8.0 | 8.4 | 8.4 | 8.6 |
|  | Diff | 0.1 | 0.1 | -0.1 | 0.0 | 0.1 | 0.1 | 0.2 | 0.2 |
| *d_50_ (cm)* | TB-10MV | 16.6 | 16.9 | 17.5 | 18.0 | 18.4 | 19.7 | 20.4 | 20.9 |
|  | E_syn_-10MV | 16.6 | 16.9 | 17.4 | 17.9 | 18.3 | 19.5 | 20.2 | 20.7 |
|  | Diff | 0.0 | 0.0 | 0.1 | 0.1 | 0.1 | 0.2 | 0.2 | 0.2 |

1. **The *RMSE*/Gamma for Wedge field.**

Table S2. The RMSE of PDDs and OCRs between TB-10 MV and E_syn_-10MV in 30° wedge field on the same Varian Truebeam Linac. (Unit: %).

| Square Field Size  （mm） | 40 | 100 | 200 | 300 | 400^a^ |
| --- | --- | --- | --- | --- | --- |
| *RMSE_PDD_* | 0.54 | 0.63 | 0.79 | 0.97 | 1.07 |
| *RMSE_OCR_* | 0.18 | 0.26 | 0.36 |  | 0.49 |

a: The field size of 400 representatives X = 300 mm and Y = 400 mm in wedge field.

Table S3. 1D-Gamma passing rate (%) for PDD and OCR between TB-10 MV and E_syn_-10MV of 2%/1mm in 30°wedge filed on the same Linacs.

|  |  | Square field size (mm) | | | | | |
| --- | --- | --- | --- | --- | --- | --- | --- |
| Type | Depth (mm) | 40 | 100 | 200 | 300 | 400 ^a^ |  |
| OCR | 50 | 100.0 | 100.0 | 100.0 | 100.0 | 100.0 |  |
|  | 100 | 100.0 | 100.0 | 100.0 | 100.0 | 100.0 |  |
|  | 200 | 100.0 | 100.0 | 100.0 | 100.0 | 100.0 |  |
|  | 300 | 100.0 | 100.0 | 100.0 | 92.2 | 90.6 |  |
| PDD |  | 99.7 | 98.0 | 99.7 | 99.7 | 99.7 |  |

a: The field size of 400 representatives X = 300 mm and Y = 400 mm in wedge field.

**3. The *RMSE*/Gamma for 10 MV energy synthesis between Varian TrueBeam and Varian** **Clinic 2300iX Linacs.**

Table S4. The RMSE of PDDs and OCRs between TB-10 MV and E_syn_-10MV for Linacs from same manufacturers. (Unit: %).

| Square Field Size  （mm） |  | 30 | 40 | 60 | 80 | 100 | 200 | 300 | 400 |
| --- | --- | --- | --- | --- | --- | --- | --- | --- | --- |
| 10-MV | *RMSE_PDD_* | 0.82 | 0.90 | 0.74 | 0.50 | 0.48 | 0.66 | 0.79 | 0.54 |
|  | *RMSE_OCR_* | 0.24 | 0.15 | 0.24 | 0.28 | 0.34 | 0.45 | 0.58 | 0.49 |

Table S5. 1D-Gamma passing rate (%) for PDD and OCR between TB-10 MV and E_syn_-10MV of 2%/1mm for Linacs from same manufacturers. (Unit: %).

|  |  | Square field size (cm) | | | | | | | |
| --- | --- | --- | --- | --- | --- | --- | --- | --- | --- |
| Type | Depth (mm) | 30 | 40 | 60 | 80 | 100 | 200 | 300 | 400* |
| OCR | 50 | 100.0 | 100.0 | 100.0 | 100.0 | 100.0 | 100.0 | 100.0 | 91.6 |
|  | 100 | 100.0 | 100.0 | 100.0 | 100.0 | 100.0 | 100.0 | 100.0 | 95.8 |
|  | 200 | 100.0 | 100.0 | 100.0 | 100.0 | 100.0 | 100.0 | 100.0 | - |
|  | 300 | 86.5 | 100.0 | 100.0 | 100.0 | 100.0 | 100.0 | 96.2 | - |
| PDD |  | 100.0 | 100.0 | 100.0 | 100.0 | 100.0 | 100.0 | 99.7 | 99.7 |

* Due to the missing OCR beam data of 400 ×400 mm2 filed size at 100, 200 mm and 300 mm depth.

**4. The RMSE/Gamma for 10 MV energy synthesis between Elekta Infinity and Varian TrueBeam Linacs.**

Table S6. The RMSE of PDDs and OCRs between ELEKTA-10 MV and E_syn_-10MV for Linacs from different manufacturers. (Unit: %).

| Square Field Size  （mm） |  | 30 | 40 | 60 | 80 | 100 | 200 | 300 | 400 |
| --- | --- | --- | --- | --- | --- | --- | --- | --- | --- |
| 10-MV | *RMSE_PDD_* | 0.47 | 0.46 | 0.44 | 0.47 | 0.43 | 0.33 | 0.40 | 0.50 |
|  | *RMSE_OCR_* | 0.76 | 0.67 | 0.62 | 0.57 | 0.51 | 0.43 | 0.59 | 0.35 |

Table S7. 1D-Gamma passing rate (%) for PDD and OCR between ELEKTA-10 MV and E_syn_-10MV of 2%/1mm for Linacs from different manufacturers. (Unit: %).

|  |  | Square field size (mm) | | | | | | | |
| --- | --- | --- | --- | --- | --- | --- | --- | --- | --- |
| Type | Depth (mm) | 30 | 40 | 60 | 80 | 100 | 200 | 300 | 400* |
| OCR | 50 | 92.4 | 93.0 | 91.5 | 93.5 | 100.0 | 100.0 | 100.0 | 100.0 |
|  | 100 | 86.6 | 94.4 | 97.6 | 98.9 | 98.1 | 100.0 | 100.0 | NULL |
|  | 200 | 89.7 | 90.5 | 91.9 | 93.9 | 94.5 | 100.0 | 99.6 | NULL |
|  | 300 | 85.5 | 90.8 | 92.1 | 94.1 | 99.1 | 100.0 | 100.0 | NULL |
| PDD |  | 100.0 | 100.0 | 100.0 | 100.0 | 99.7 | 100.0 | 100.0 | 100.0 |

* Due to the missing OCR beam data of 400 x 400 mm^2^ filed size at 100, 200 mm and 300 mm depth.

**5. The photon energy synthesis of BJR data.**


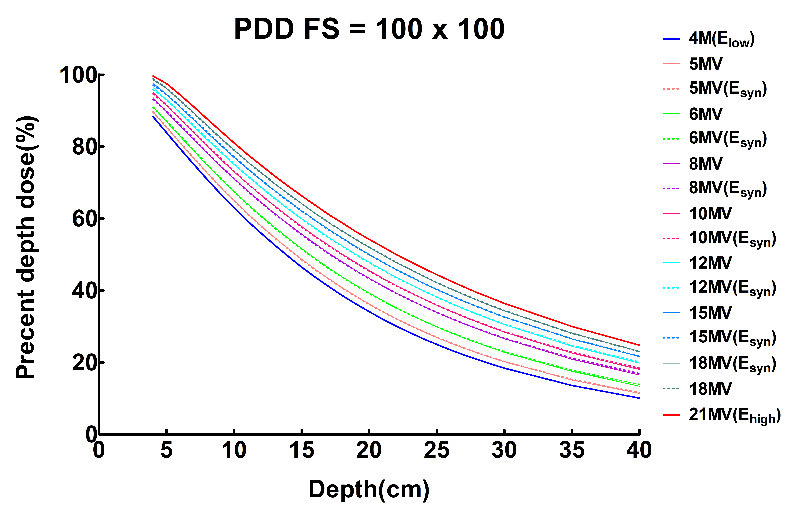


**(A)**


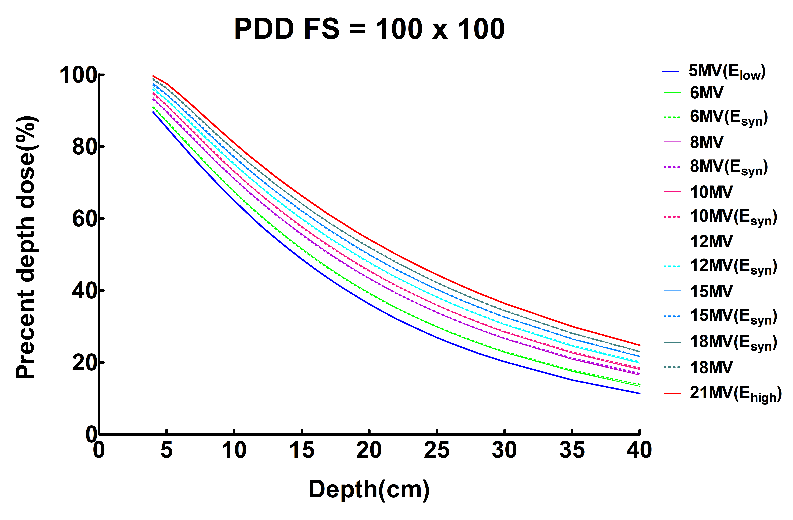


**(B)**


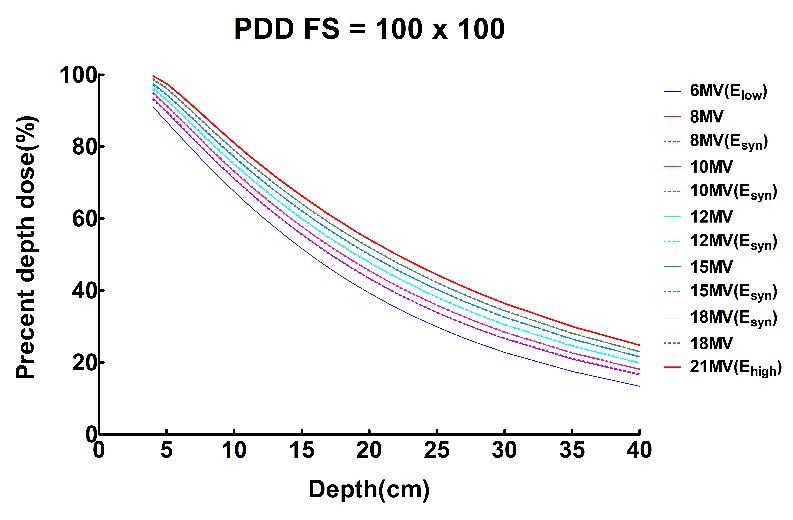


**(C)**


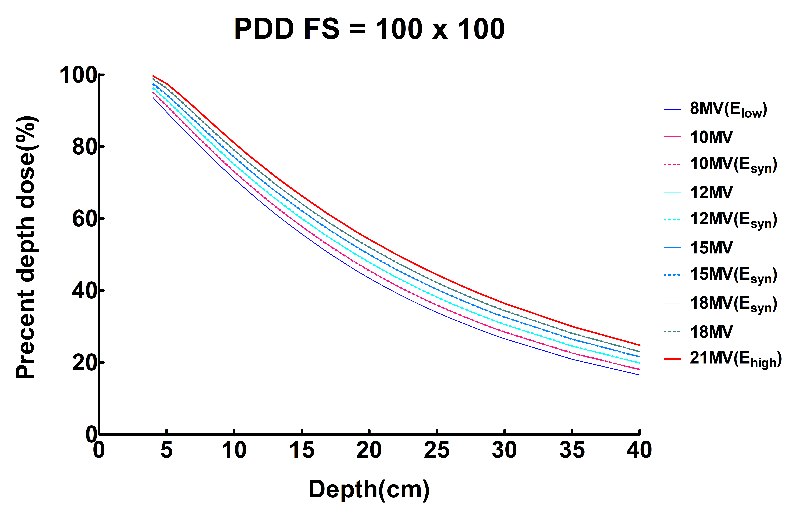


**(D)**


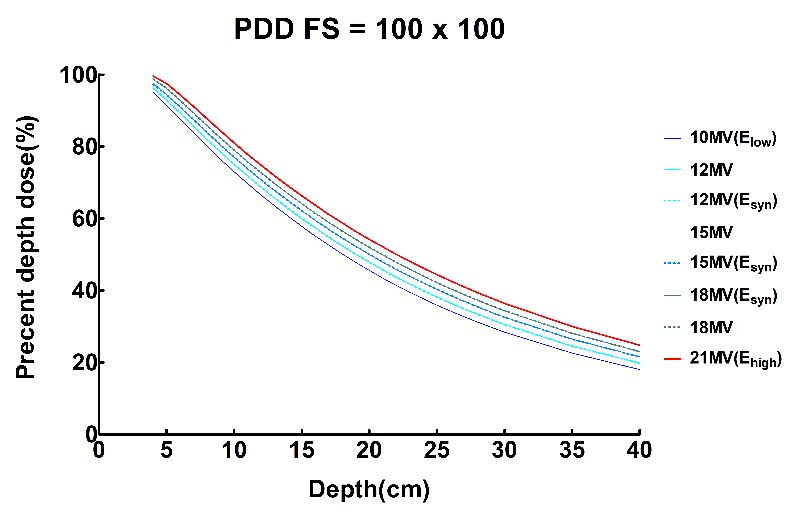


**(E)**


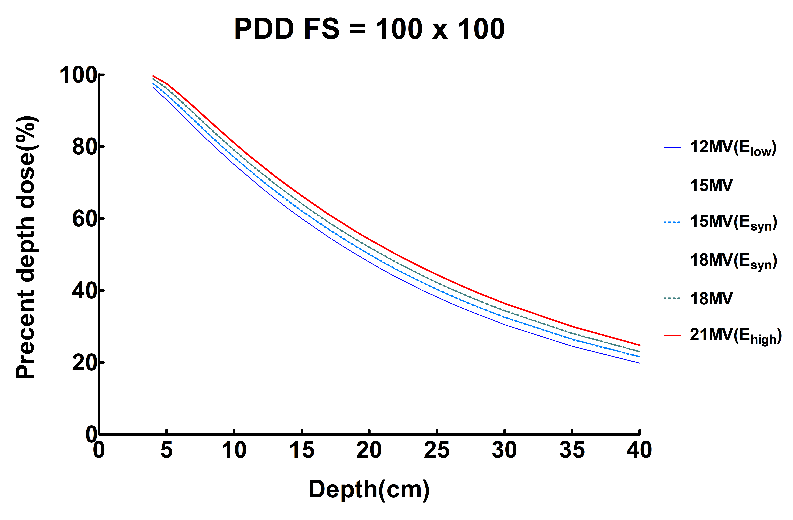


**(F)**


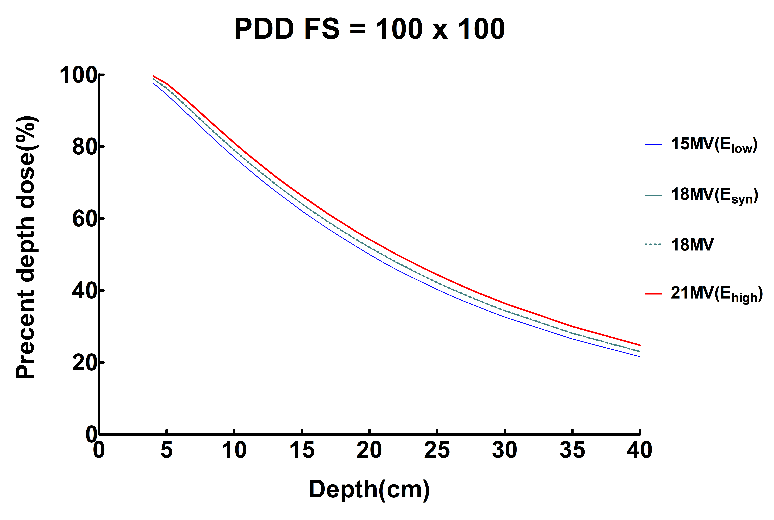


**(G)**

Figure S1. The differences of PDDs between *E_mid_* and *E_syn_* for the 100 × 100 mm^2^ field size with *E_high_* was fixed at 21 MV as and when *E_low_* was changed to (A) 4 MV, (B) 5 MV, (C) 6 MV, (D) 8 MV, (E) 10 MV, (F) 12 MV and (G) 15 MV.

**6. Clinical case treatment plans.**


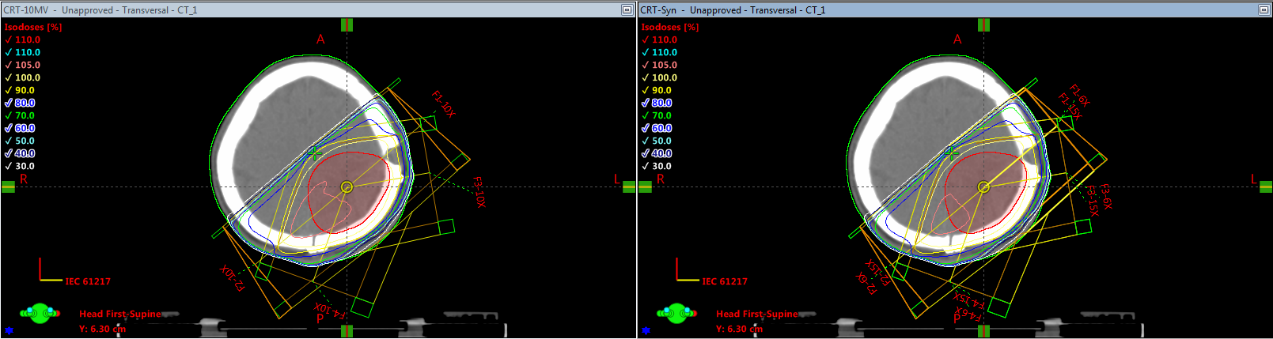


(A)


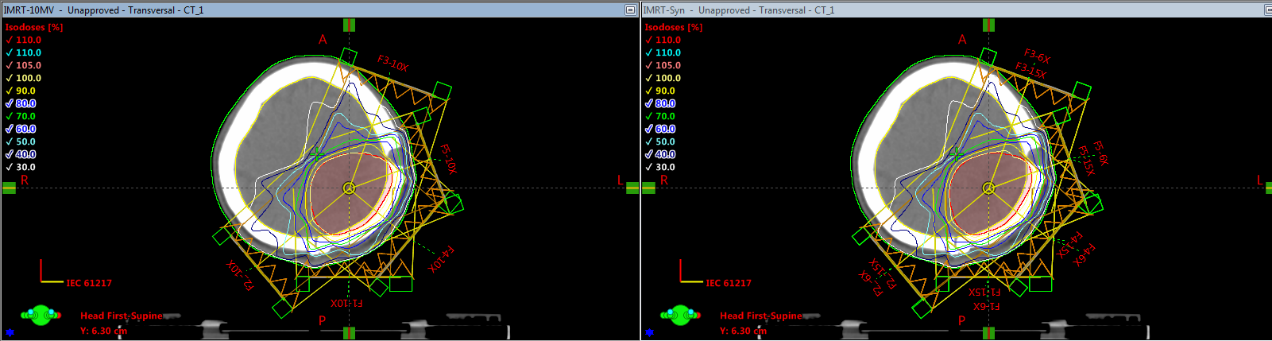
(B)


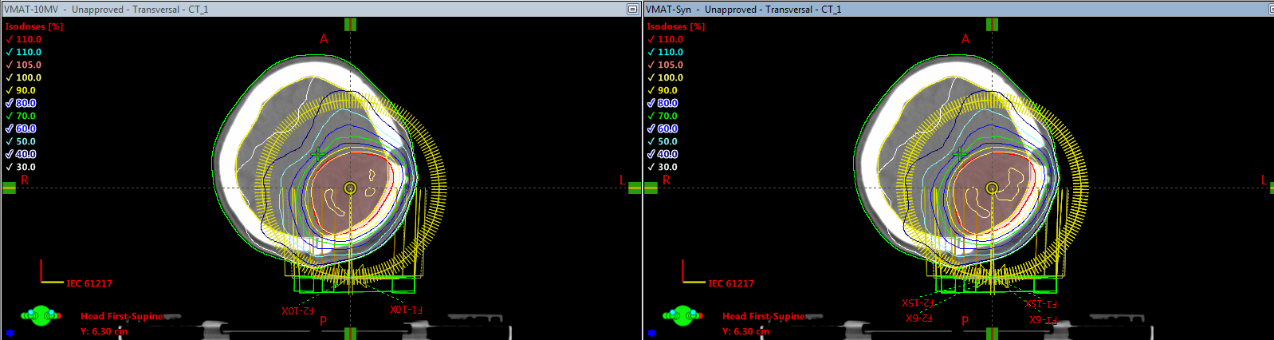


(C)

Figure S2. Comparison of dose distributions of (A)CRT, (B)IMRT and (C)VMAT plans in the intra-cranial case. (Left: TB-10MV, and Right: E_syn_-10MV)


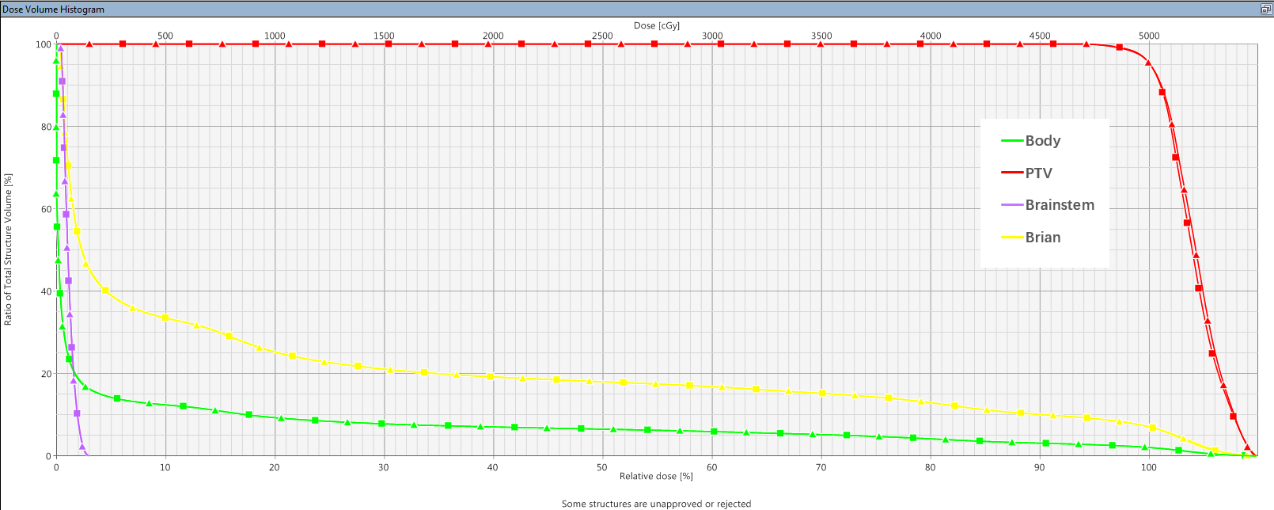


(A)
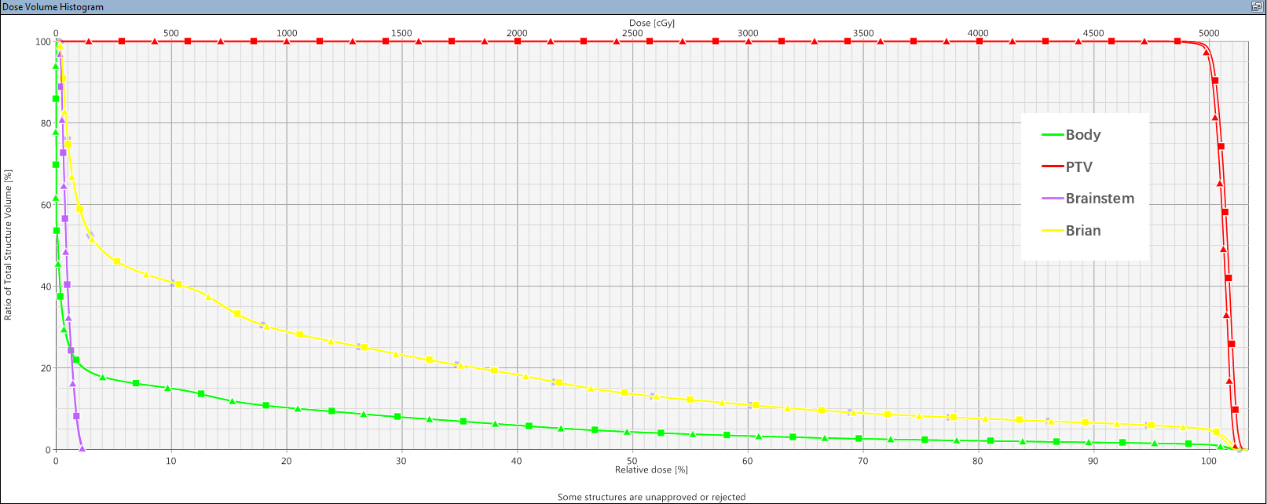


(B)
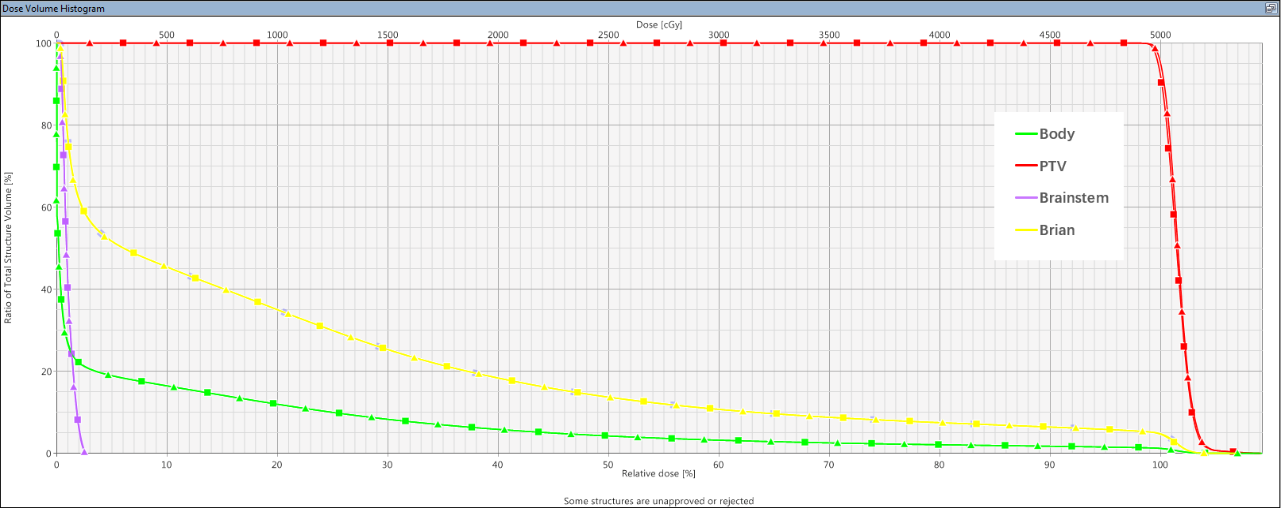


(C)

Figure S3. Dose-volume histograms of two corresponding (A)CRT, (B)IMRT and (C)VMAT plans in the liver case. (Square solid line: TB-10MV; Triangle solid line: E_syn_-10MV)


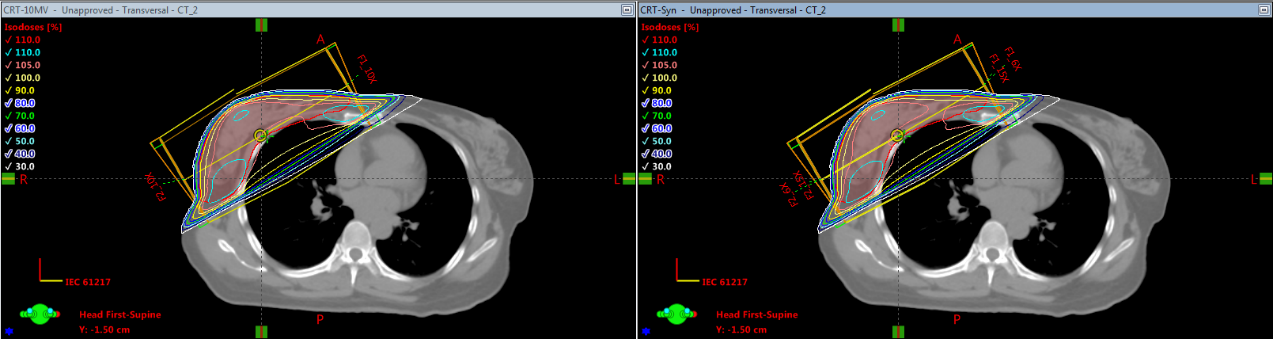
(A)


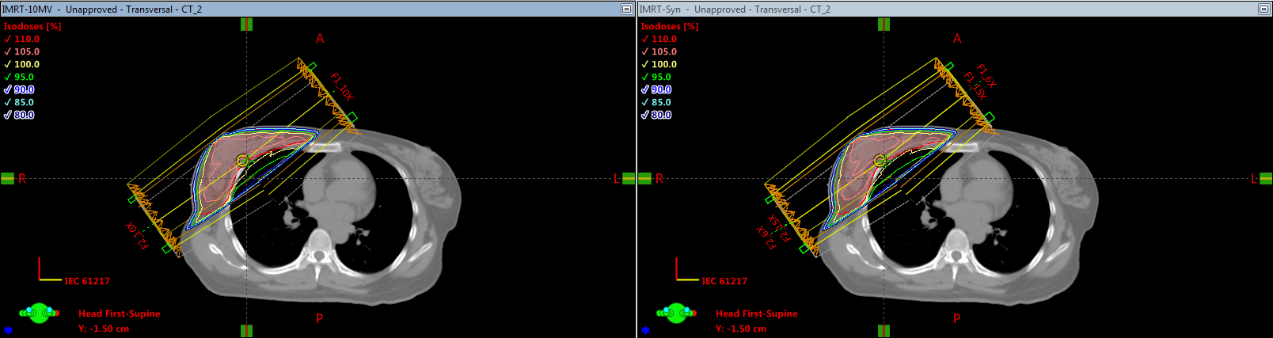
(B)


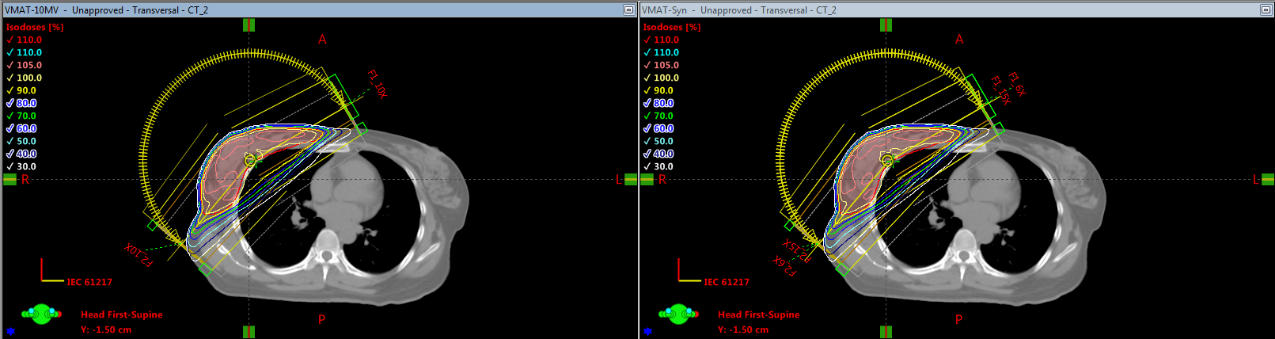
(C)

Figure S4. Comparison of dose distributions of two (A)CRT, (B)IMRT and (C)VMAT plans in the breast case. (Left: TB-10MV, and Right: E_syn_-10MV)


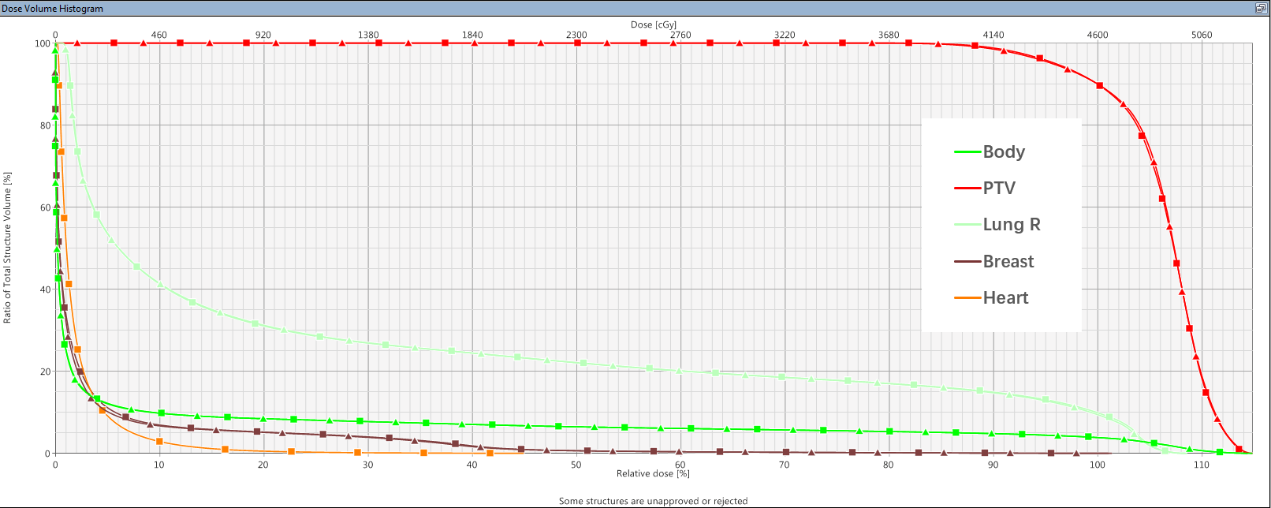
(A)


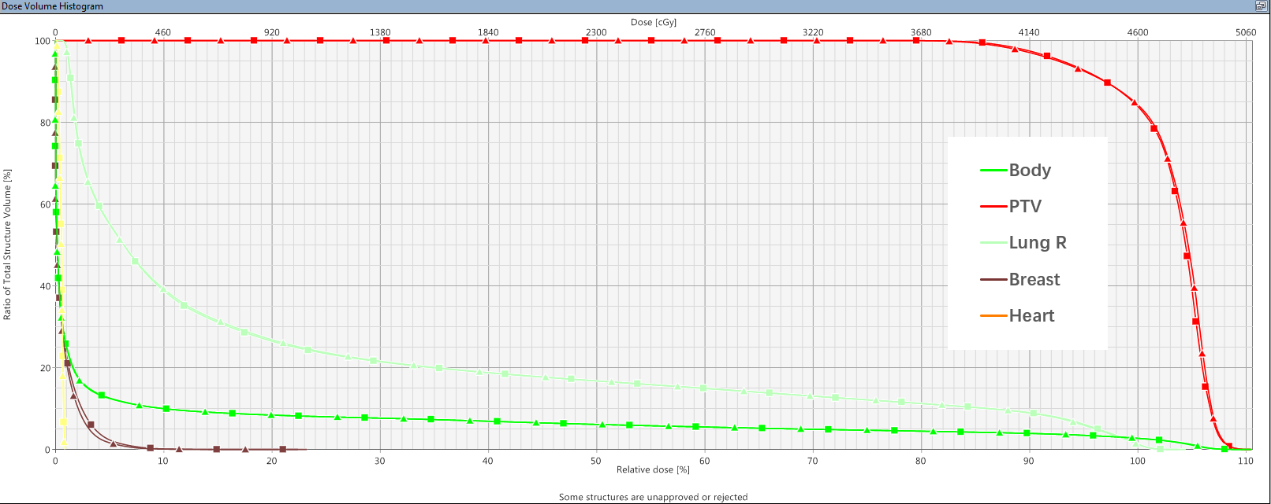
(B)


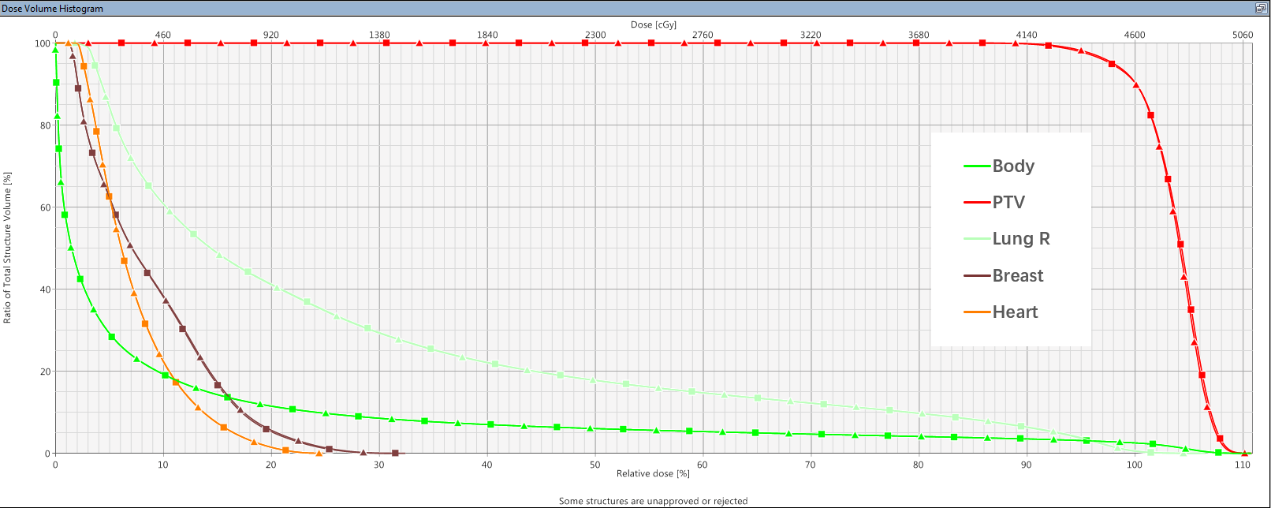
(C)

Figure S5. Dose-volume histograms of two corresponding (A)CRT, (B)IMRT and (C)VMAT plans in the breast case. (Square solid line: TB-10MV; Triangle solid line: E_syn_-10MV)


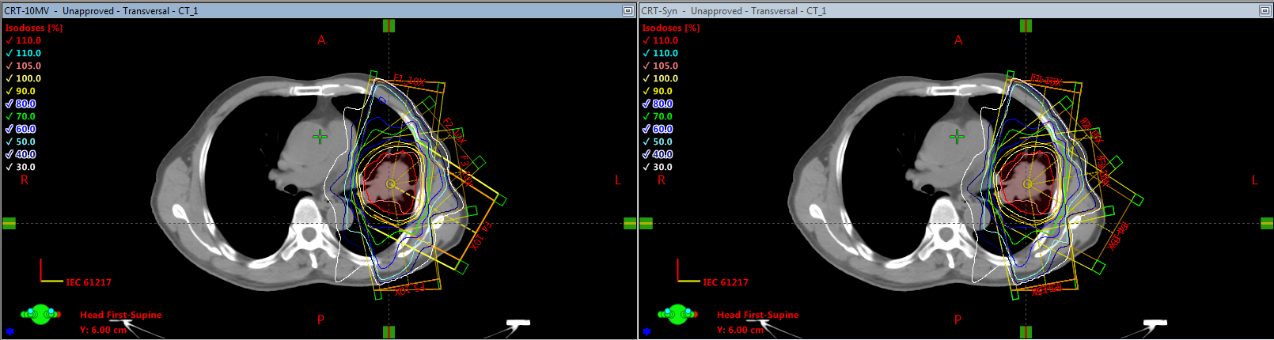
(A)


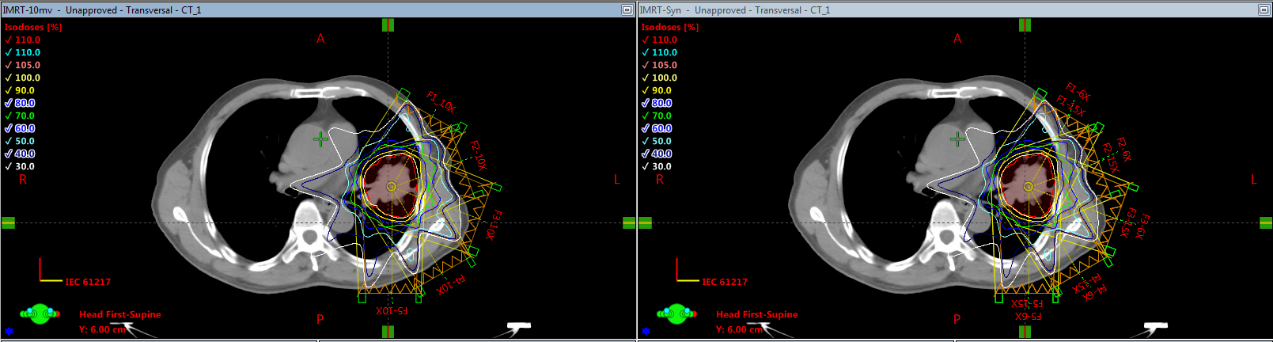


(B)


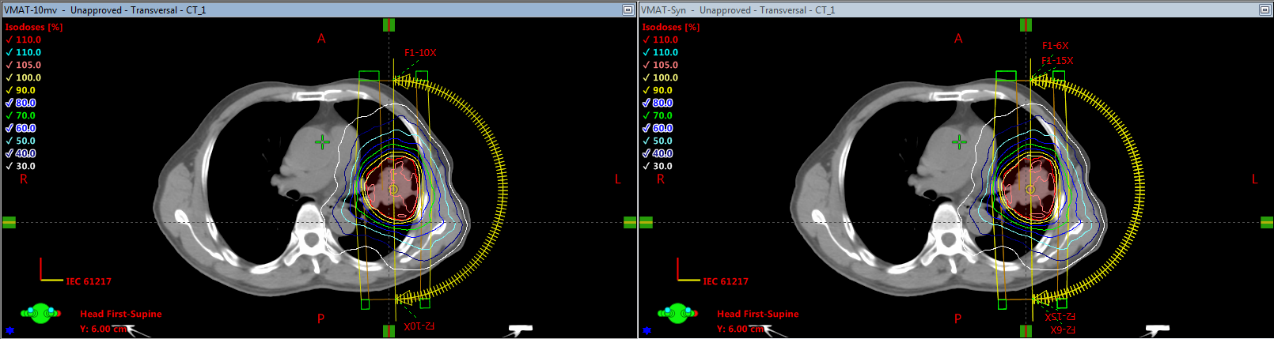
(C)

Figure S6. Comparison of dose distributions of two (A)CRT, (B)IMRT and (C)VMAT plans in the lung case. (Left: TB-10MV, and Right: E_syn_-10MV)


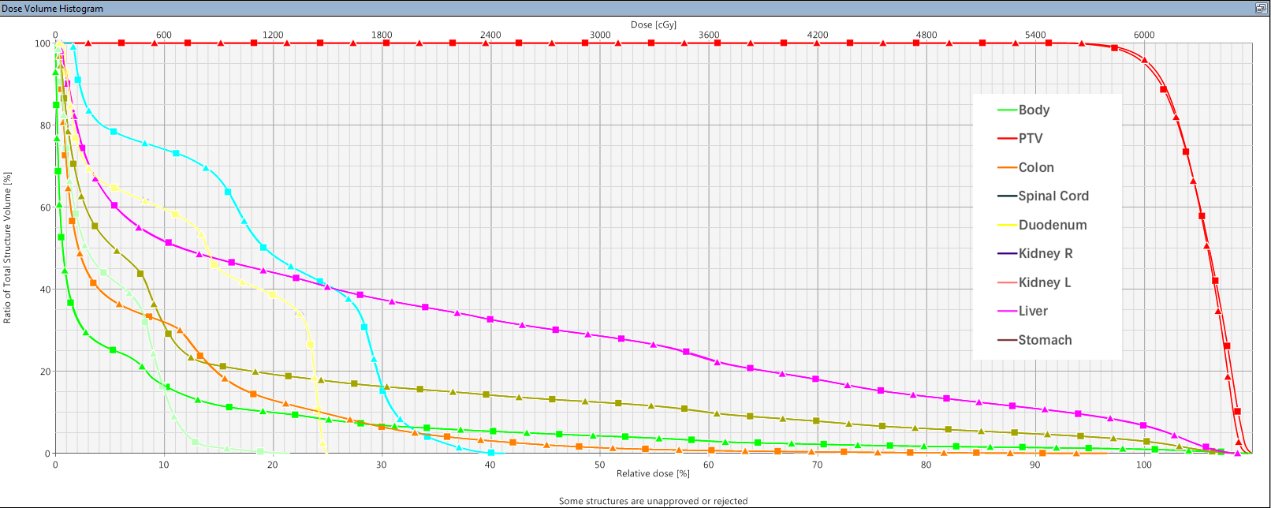


(A)
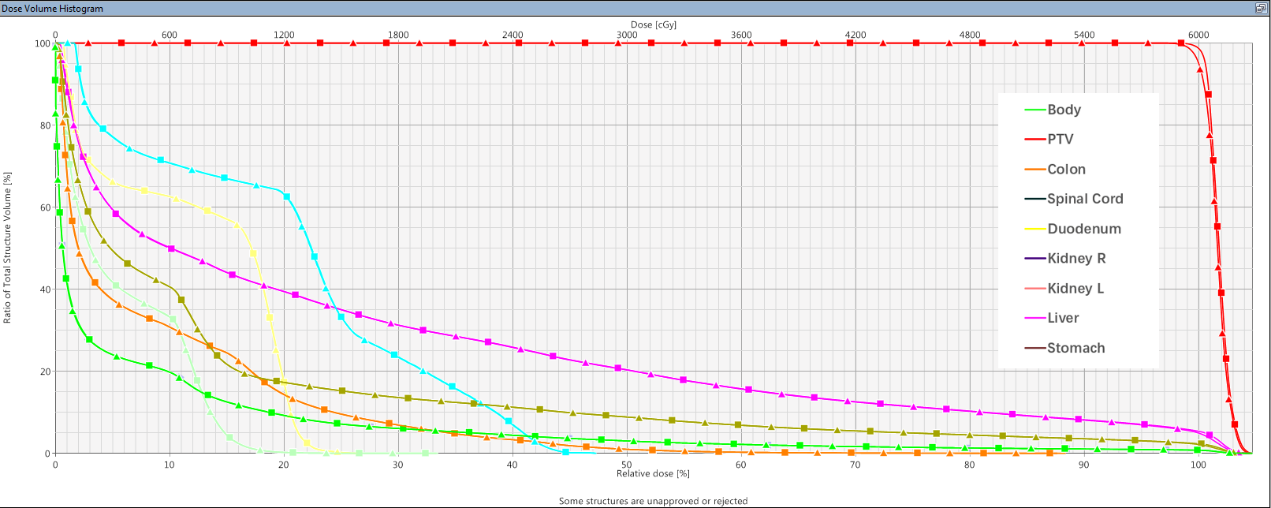


(B)
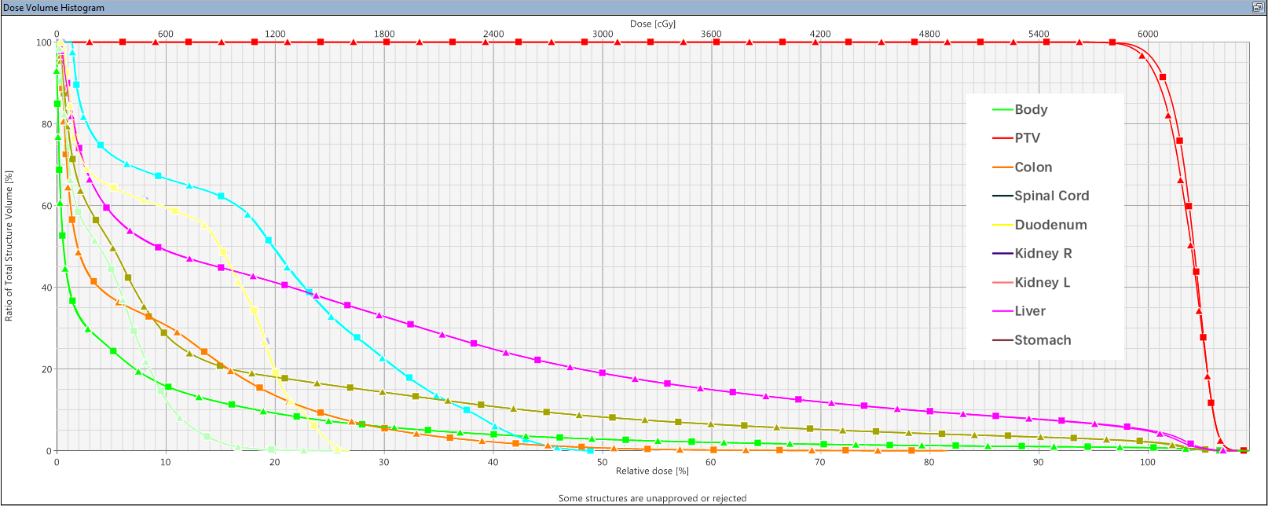


(C)

Figure S7. Dose-volume histograms of two corresponding (A)CRT, (B)IMRT and (C)VMAT plans in the lung case. (Square solid line: TB-10MV; Triangle solid line: E_syn_-10MV)


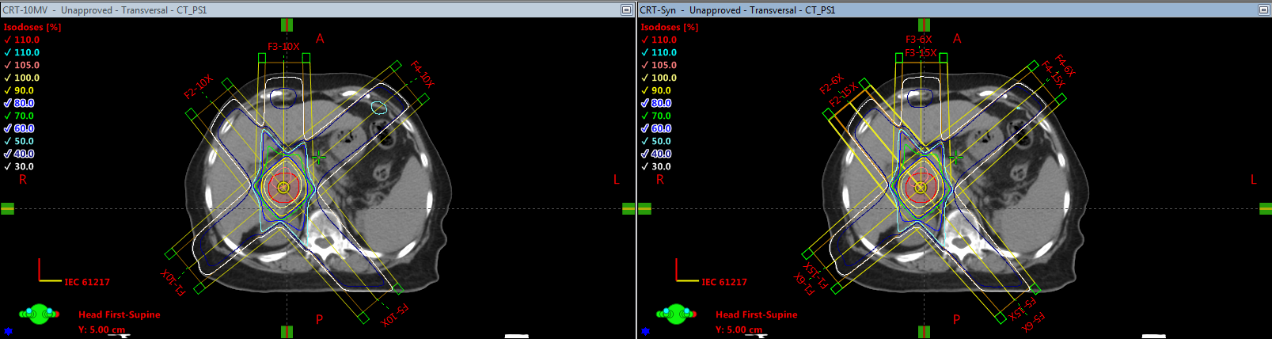
(A)


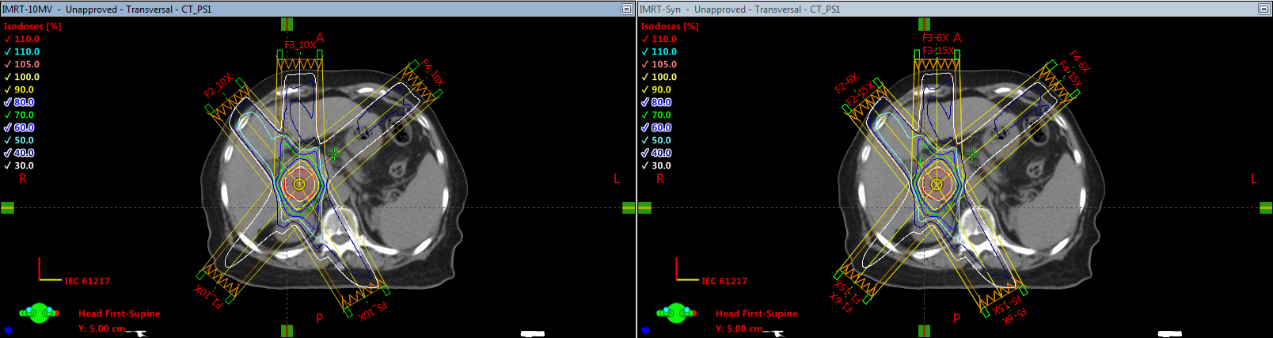
(B)
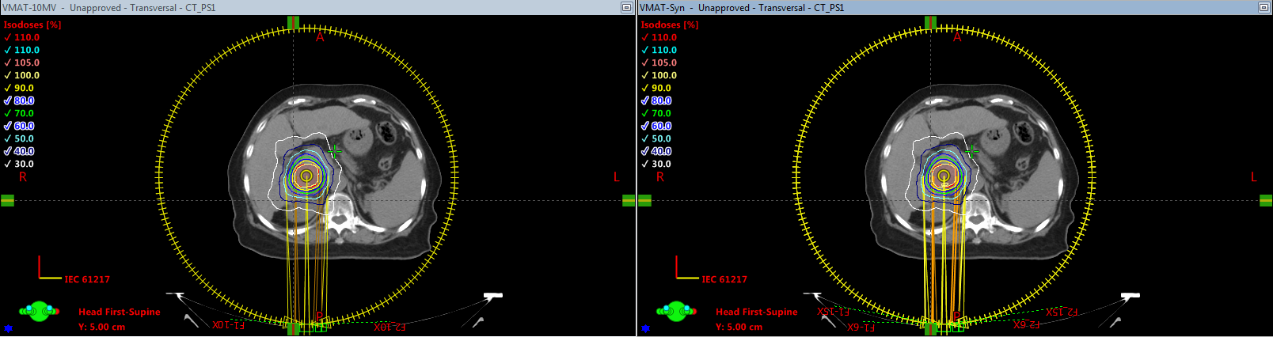
 (C)

Figure S8. Comparison of dose distributions of two (A)CRT, (B)IMRT and (C)VMAT plans in the liver case. (Left: TB-10MV, and Right: E_syn_-10MV)


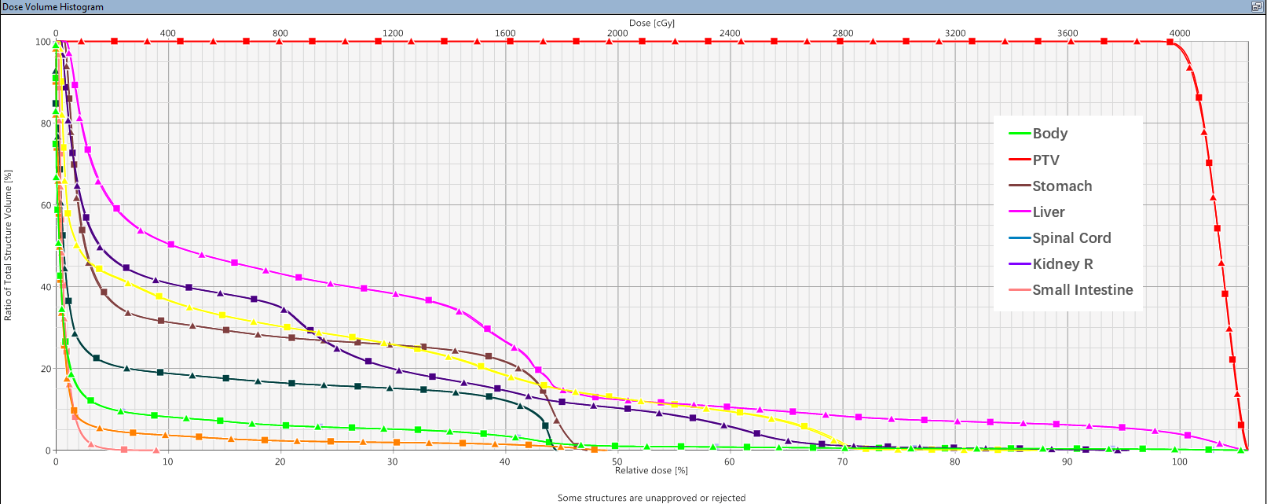
(A)


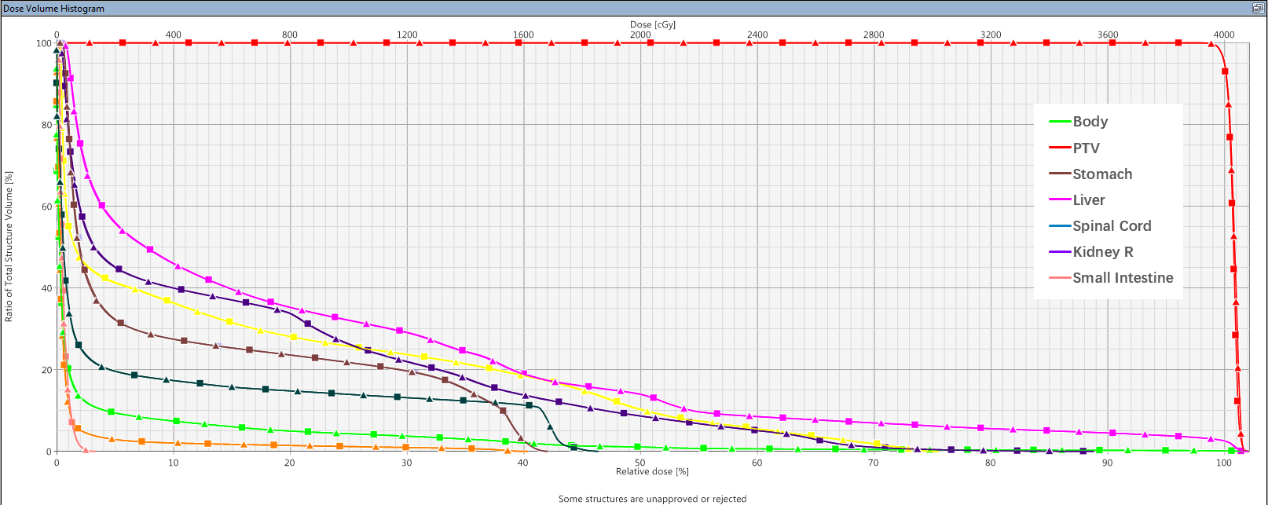
(B)


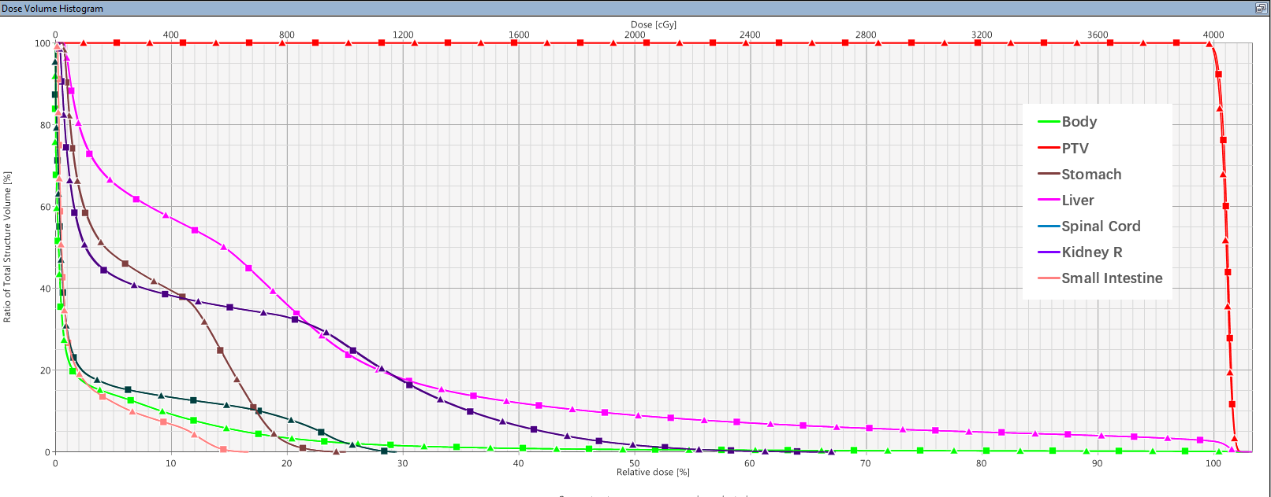
(C)

Figure S9. Dose-volume histograms of two corresponding (A)CRT, (B)IMRT and (C)VMAT plans in the liver case. (Square solid line: TB-10MV; Triangle solid line: E_syn_-10MV)


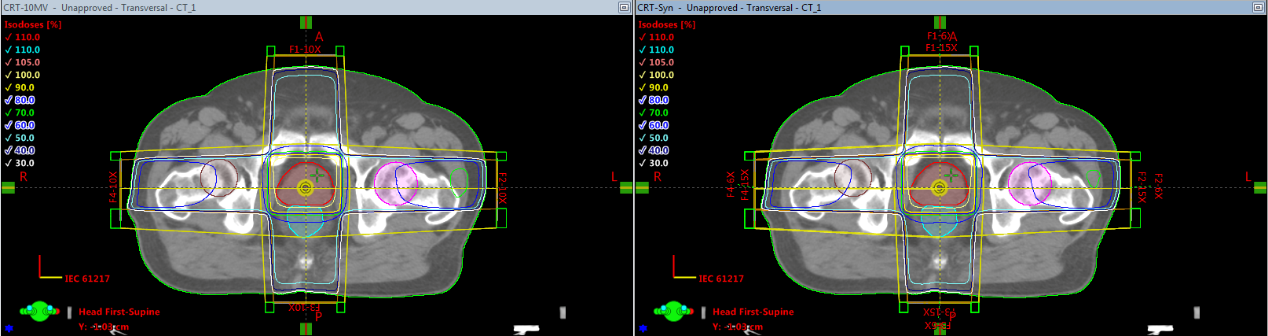
(A)


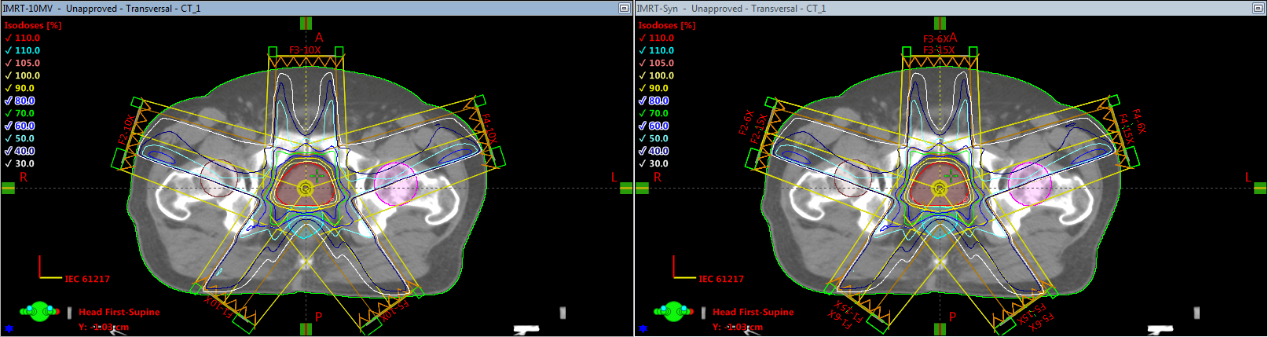
(B)
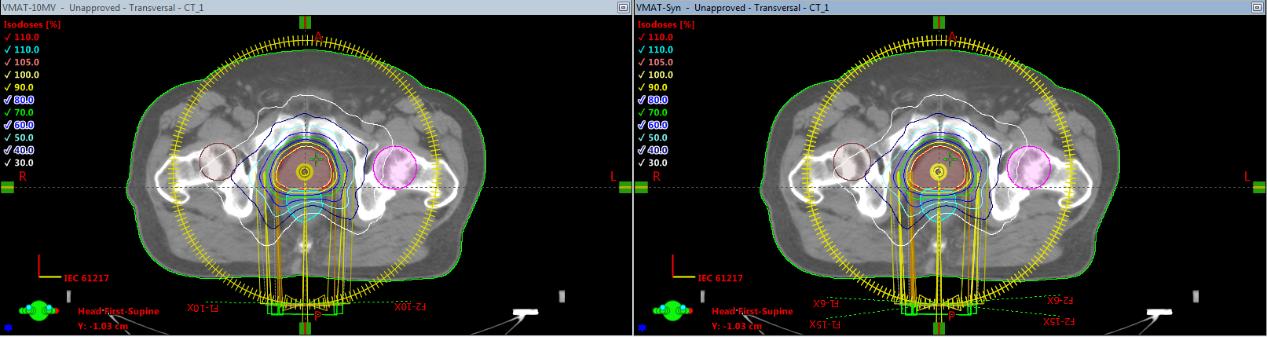
 (C)

Figure S10. Comparison of dose distributions of two (A)CRT, (B)IMRT and (C)VMAT plans in the prostate case. (Left: TB-10MV, and Right: E_syn_-10MV)


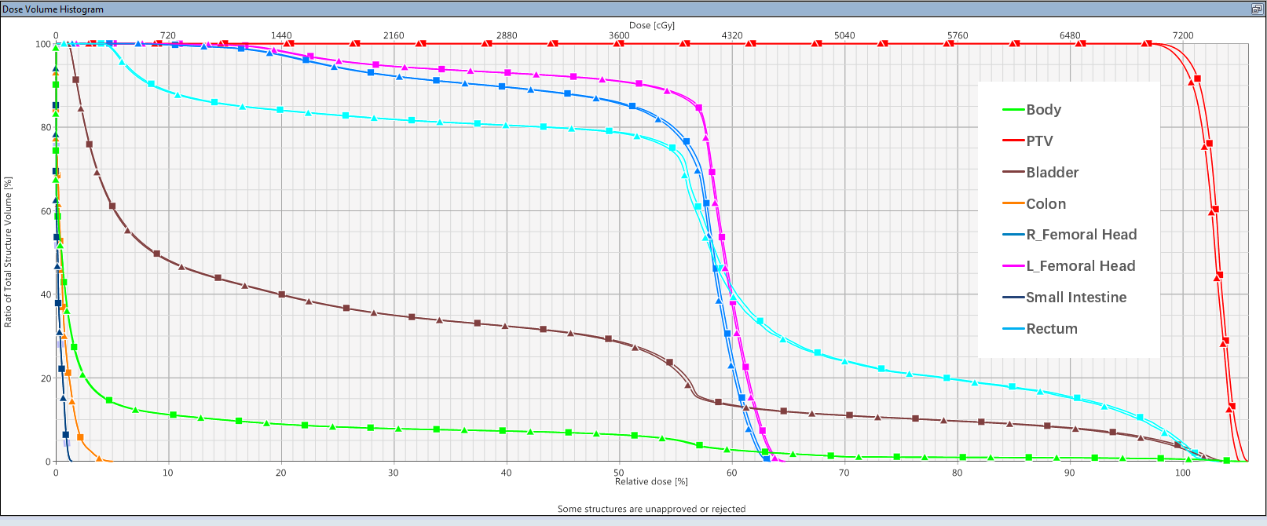
(A)


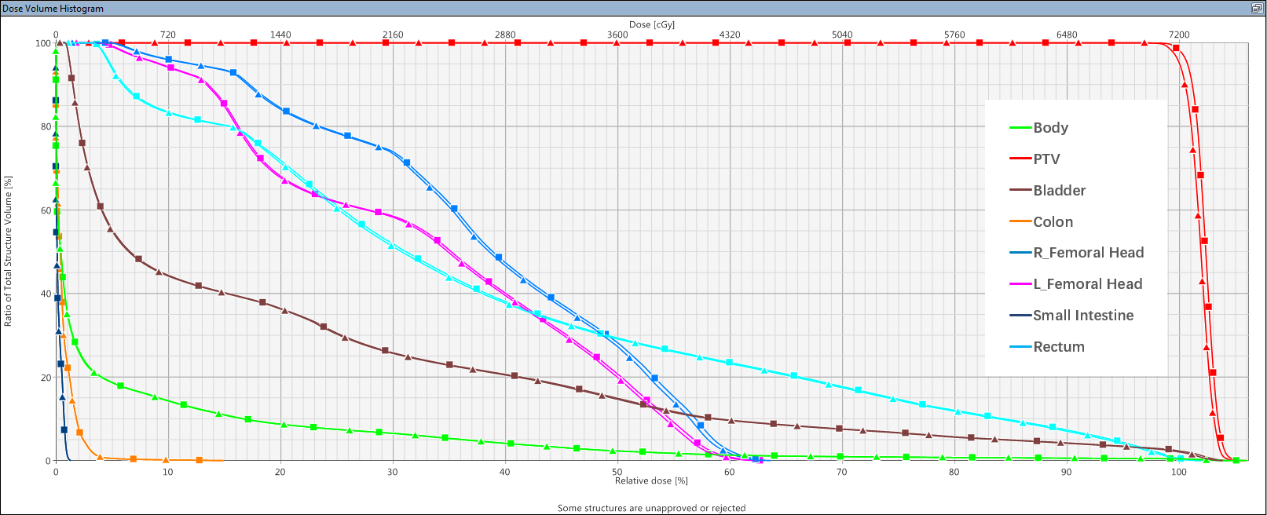
(B)


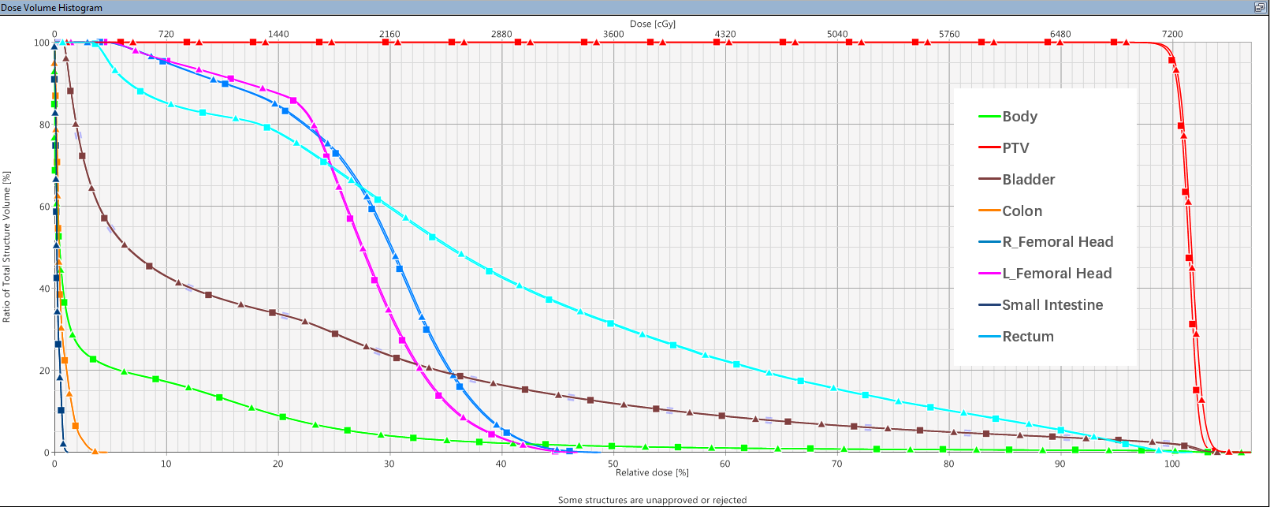
(C)

Figure S11. Dose-volume histograms of two corresponding (A)CRT, (B)IMRT and (C)VMAT plans in the prostate case. (Square solid line: TB-10MV; Triangle solid line: E_syn_-10MV)
